# DNA Copy Number Changes in Human Malignant Fibrous Histiocytomas by Array Comparative Genomic Hybridisation

**DOI:** 10.1371/journal.pone.0015378

**Published:** 2010-11-09

**Authors:** Stine H. Kresse, Hege O. Ohnstad, Bodil Bjerkehagen, Ola Myklebost, Leonardo A. Meza-Zepeda

**Affiliations:** 1 Department of Tumour Biology, The Norwegian Radium Hospital, Oslo University Hospital, Oslo, Norway; 2 Pathology Clinic, The Norwegian Radium Hospital, Oslo University Hospital, Oslo, Norway; 3 Norwegian Microarray Consortium, Department of Molecular Biosciences, University of Oslo, Oslo, Norway; Health Canada, Canada

## Abstract

**Background:**

Malignant fibrous histiocytomas (MFHs), or undifferentiated pleomorphic sarcomas, are in general high-grade tumours with extensive chromosomal aberrations. In order to identify recurrent chromosomal regions of gain and loss, as well as novel gene targets of potential importance for MFH development and/or progression, we have analysed DNA copy number changes in 33 MFHs using microarray-based comparative genomic hybridisation (array CGH).

**Principal findings:**

In general, the tumours showed numerous gains and losses of large chromosomal regions. The most frequent minimal recurrent regions of gain were 1p33-p32.3, 1p31.3-p31.2 and 1p21.3 (all gained in 58% of the samples), as well as 1q21.2-q21.3 and 20q13.2 (both 55%). The most frequent minimal recurrent regions of loss were 10q25.3-q26.11, 13q13.3-q14.2 and 13q14.3-q21.1 (all lost in 64% of the samples), as well as 2q36.3-q37.2 (61%), 1q41 (55%) and 16q12.1-q12.2 (52%). Statistical analyses revealed that gain of 1p33-p32.3 and 1p21.3 was significantly associated with better patient survival (*P* = 0.021 and 0.046, respectively). Comparison with similar array CGH data from 44 leiomyosarcomas identified seven chromosomal regions; 1p36.32-p35.2, 1p21.3-p21.1, 1q32.1-q42.13, 2q14.1-q22.2, 4q33-q34.3, 6p25.1-p21.32 and 7p22.3-p13, which were significantly different in copy number between the MFHs and leiomyosarcomas.

**Conclusions:**

A number of recurrent regions of gain and loss have been identified, some of which were associated with better patient survival. Several specific chromosomal regions with significant differences in copy number between MFHs and leiomyosarcomas were identified, and these aberrations may be used as additional tools for the differential diagnosis of MFHs and leiomyosarcomas.

## Introduction

The concept of malignant fibrous histiocytoma (MFH) has changed during the last decades, and is now used to describe a heterogeneous group of tumours without a specific known lineage of differentiation and with fibroblastic/myofibroblastic features. Tumours still classified as MFHs are also termed undifferentiated high grade pleomorphic sarcomas (UPSs) according to the latest World Health Organization (WHO) classification [1]. MFHs were initially assigned to several subgroups; pleomorphic, myxoid, giant cell and inflammatory, which are still used. However, several so-called giant cell MFHs are now reclassified as other giant cell sarcomas, and several so-called inflammatory MFHs are now recognized to be dedifferentiated liposarcomas [2]. The so-called myxoid MFHs are now termed myxofibrosarcomas [1], and this second common subtype have a better prognosis than the most common subtype, pleomorphic MFHs [3].

MFHs occur mainly late in life, between the age of 50 and 70 years, and the main locations are in the lower extremities followed by the upper extremities and the retroperitoneum [3]. The main exception is inflammatory MFHs, which are most often located in the retroperitoneum. MFHs were previously regarded as the most common soft tissue sarcoma of adults, and depending on the criteria used for classification, MFHs still account for a considerable portion of these tumours. Men are more frequently affected than women, about 2/3 of the tumours occur in men [3]. MFHs are in general high-grade tumours, and the 5-year survival is 65–70% [3].

Cytogenetic studies have revealed that most MFHs have complex karyotypes with numerous aberrations, both numerical and structural. Using chromosome-based comparative genomic hybridization (CGH), recurrent gains of regions in 1p, 1q, 5p, 17p, 17q and 20q have frequently been observed, as well as losses of regions in 2q, 9p, 10q, 11q and 13q [4], [5], [6], [7], [8], [9], [10], [11]. High-level amplification of the distal part of 13q has frequently been found [5], [6]. Gain of 17q has been associated with longer disease-free survival and low risk of developing distant metastasis [9], whereas gain of 7q32 has been associated with poor prognosis [5]. In addition, patients with gain of 1p31 showed a trend towards decreased overall survival [5].

More recently, microarray-based CGH (array CGH) has been used to analyse DNA copy number changes at higher resolution. In order to identify specific genomic events and candidate targets that may play a role in MFH development and/or progression, we have used array CGH to map the distribution and frequency of DNA copy number changes at high resolution in 33 MFH samples. Statistical analyses were performed in order to identify possible correlations between the experimental results and the clinical information. In addition, the results were compared to array CGH data from 44 leiomyosarcomas in order to identify chromosomal aberrations significantly different between the two tumour types, since their differential diagnosis may be difficult due to histological similarities.

## Materials and Methods

### Tumour samples

Thirty-one human sarcomas classified as MFHs were selected from a tumour collection at the Department of Tumour Biology at The Norwegian Radium Hospital. In addition, two samples initially diagnosed as leiomyosarcomas were included in the study after reclassification to MFH (MFH73x and MFH76x). All tumours were revised at the time of the study by an expert pathologist (B.B.) and diagnosed according to the current WHO classification [1].

Clinical samples were collected immediately after surgery, cut into small pieces, frozen in liquid nitrogen and stored at −70°C until use. Some of the samples were grown subcutaneously in immunodeficient mice as xenografts (suffix x). The clinical information was retrieved from the MEDinsight database at The Norwegian Radium Hospital. Clinical data for all samples are given in Table 1.

**Table 1:** Clinical data of malignant fibrous histiocytomas.

Sample	Sample origin	Patient age/sex	Diagnosis	MFH Subtype	Grade^1^	Primary tumour location	Size (cm)^2^	Metastasis (months)^3^	Status	Follow-up (months)^4^
MFH1	Rec	87/M	MFH	Myxofibrosarcoma	4	Lower arm	5	48	DD	50
MFH2x	Rec	71/M	MFH	Spindle and pleomorphic	4	Upper trunk	19	4	DD	15
MFH4	Prim	77/M	MFH	Spindle and pleomorphic	4	Upper arm	15	MD	DD	3
MFH7	Prim	68/M	MFH	Spindle and pleomorphic	4	Upper leg	6	NM	DOC	74
MFH8	Met	76/M	MFH	Spindle and pleomorphic	4	Knee^5^	6	15	DD	27
MFH9	Rec	57/F	MFH	Spindle and pleomorphic	4	Upper leg	3	NM	DOC	346
MFH14	Prim	60/M	MFH	Spindle and pleomorphic	4	Knee	5.5	38	DD	82
MFH15	Prim	45/F	MFH	Pleomorphic with giant cells	4	Upper leg	20	6	DOC	6
MFH16	Rec	63/M	MFH	Myxofibrosarcoma	4	Upper trunk	NA	NM	DOC	191
MFH18	Prim	61/M	MFH	Spindle and pleomorphic	4	Lower leg	18	16	DD	28
MFH19	Rec	71/F	MFH	Spindle and pleomorphic	4	Upper leg	5	NM	NED	168
MFH20	Met	66/M	MFH	Spindle and pleomorphic	4	Upper leg^6^	18	17	DD	40
MFH21	Prim	73/F	MFH	Spindle and pleomorphic	4	Upper trunk	15	7	DD	21
MFH24	Prim	92/F	MFH	Spindle and pleomorphic	4	Upper leg	13	NM	NED	159
MFH25	Prim	47/F	MFH	Myxofibrosarcoma	4	Upper leg	20	NM	NED	182
MFH27	Rec	60/M	MFH	Spindle and pleomorphic	3	Knee	7.5	34	DD	181
MFH30	Prim	56/M	MFH	Spindle and pleomorphic	4	Retroperitoneum	15	MD	DD	3
MFH34	Rec	79/F	MFH	Spindle and pleomorphic	3	Upper trunk	1.7	NM	DOC	80
MFH36	Prim	75/M	MFH	Myxofibrosarcoma	4	Shoulder	20	7	DD	14
MFH42	Prim	71/M	MFH	Pleomorphic with giant cells	4	Upper arm	7.5	4	DD	22
MFH44	Rec	80/F	MFH	Spindle and pleomorphic	4	Upper leg	13	NM	DOC	15
MFH45	Prim	69/F	MFH	Spindle and pleomorphic	4	Upper arm	14	NM	NED	119
MFH46	Prim	82/M	MFH	Spindle and pleomorphic	4	Upper leg	11	NM	NED	86
MFH47	Prim	56/M	MFH	Spindle and pleomorphic	4	Upper leg	7	7	DD	19
MFH48	Prim	63/M	MFH	Spindle and pleomorphic	4	Lower leg	7	MD	DD	100
MFH53	Prim	69/F	MFH	Pleomorphic with giant cells	4	Retroperitoneum	18	5	DD	10
MFH54	Prim	45/M	MFH	Myxofibrosarcoma	4	Upper leg	20	6	DD	7
MFH56	Prim	75/F	MFH	Spindle and pleomorphic	4	Upper arm	10	9	NED	85
MFH59	Prim	68/M	MFH	Spindle and pleomorphic	4	Upper trunk	10.5	MD	DD	7
MFH60	Prim	58/F	MFH	Pleomorphic	4	Upper arm	11	9	DD	58
MFH61	Prim	66/M	MFH	Myxofibrosarcoma	4	Upper leg	9.5	NM	NED	74
MFH73x	Rec	82/F	MFH	Spindle cell	4	Lower arm	7	14	DOC	59
MFH76x	Prim	69/F	MFH	Spindle and pleomorphic	4	Lower leg	10	8	DD	18

Abbreviations: x, xenograft; Rec, recurrence; Prim, primary tumour; Met: metastasis; M, male; F, female; MFH, malignant fibrous histiocytoma; NA, not available; MD, metastasis at diagnosis; NM, no metastasis; DD, dead of disease; DOC, dead of other cause; NED, no evidence of disease;

1 Grading is based on a four-tiered system used in the Scandinavian Sarcoma Group (SSG);

2 Largest diameter of the tumour;

3 Time to first metastasis from diagnosis;

4 Time to last follow-up from diagnosis;

5 Metastasis located in the lung;

6 Metastasis located in the abdominal wall.

### Ethics Statement

The information given to the patients, the written consent used, the collection of samples, and the research project were approved by the ethical committee of Southern Norway (Project S-06133). Human tumour samples were obtained with the corresponding written consent given by the patients. Animal care was in accordance with National and institutional guidelines and the project approved by the National Committee on Research on Animal Care (Project 1498).

### Array CGH

he genomic microarray used contained 4,549 bacterial- and P1 artificial chromosome (BAC and PAC) clones representing the human genome at approximately 1 Mb resolution, as well as the minimal tiling-path between 1q12 and the beginning of 1q25. Detailed information on the construction and preparation of the microarray has been previously described [12]. The microarrays were provided by the Norwegian Microarray Consortium (www.microarray.no).

Array CGH was performed essentially as described previously [12]. In brief, approximately 500 ng of *Dpn*II-digested total genomic DNA was labelled by random priming using BioPrime DNA Labeling System (Invitrogen, California, USA) and Cy3-dCTP (tumour) or Cy5-dCTP (reference) (PerkinElmer, Massachusetts, USA). Labelled tumour and reference DNA were combined together with 135 µg human Cot-1 DNA (Invitrogen). Hybridisation was performed using an automated hybridisation station, GeneTAC (Genomic Solutions/PerkinElmer), agitating the hybridisation solution for 42–46 hours at 37°C. The arrays were scanned using an Agilent G2565BA scanner (Agilent Technologies, California, USA), and the images were segmented using GenePix Pro 6.0 (Axon Laboratories, California, USA). Further data processing, including filtering and normalization, was performed using M-CGH as previously described [12], [13].

### Array CGH data analysis

The complete array CGH dataset for the 33 MFHs can be viewed in the ArrayExpress microarray database (www.ebi.ac.uk/arrayexpress, accession number E-MEXP-1804). Clones belonging to chromosomes 1–22 with known unique chromosomal location in Ensembl (www.ensembl.org, v33, Sep 2005) were considered for analysis (3,351 clones). Due to experimental variation in normal control experiments, 22 clones (0.7%) were discarded as described previously [12]. In addition, clones with missing values in 10 or more of the 33 samples were discarded, leaving 3,144 clones for analysis. The remaining missing values were imputed via a K-Nearest Neighbour algorithm normalization using “Significance Analysis of Microarrays” (SAM) [14].

Clustering of all samples was performed using J-Express v. 2.7 [15], with average linkage (WPGMA) as the cluster method and Pearson correlation as the distance metric. In order to determine copy number changes, CGH-Explorer v. 3.1b was used [16]. “Analysis of Copy Errors” (ACE) was performed using a false discovery rate of 0.0001 and medium sensitivity. Chromosomal segments showing gains or losses in at least 10 of 33 MFHs (>30%) were used to identify minimal recurrent regions of alteration.

### Statistical analysis

Statistical analyses were performed using SPSS 15.0. Samples were categorized based on the experimental results and compared with the clinical data (Table 1). Overall survival was analyzed using Kaplan-Meier survival curves and tested for significance using the Log Rank test for all clinical variables and minimal recurrent chromosomal regions altered. *P* values less than or equal to 0.05 were considered to be statistically significant. Multivariate analysis of experimental and clinical variables significantly associated with survival was performed using Cox regression.

In order to identify chromosomal regions with significantly different DNA copy number between two groups of samples, a two-class unpaired t-test was performed using SAM [14]. Using 100 permutations and a false discovery rate of <1%, a list of genomic clones showing significant copy number differences was generated. Chromosomal segments represented by multiple significant clones (at least five significant clones with less than 10 non-significant clones between two significant clones) were considered to be significantly different between the two groups.

## Results

### Recurrently altered chromosomal regions in malignant fibrous histiocytomas

**Figure 1:**
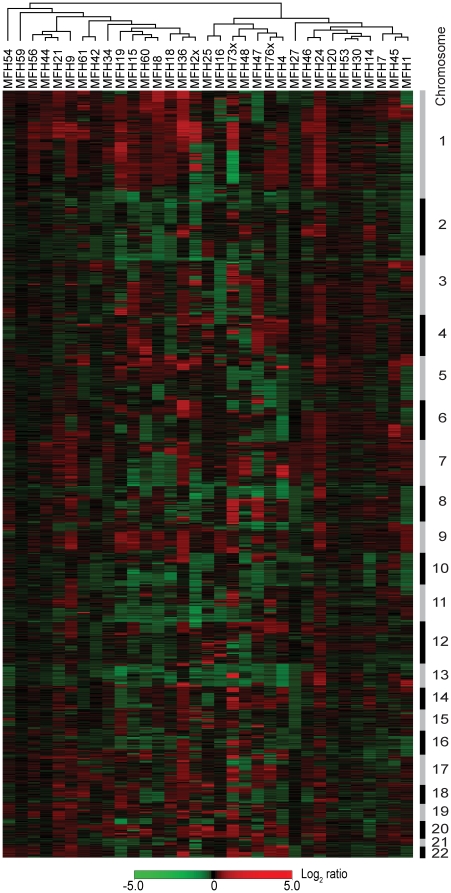
Hierarchical clustering of MFHs. Hierarchical clustering of 33 MFHs using DNA copy number ratios relative to a pool of normal diploid DNA. A total of 3,144 unique genomic clones are shown in chromosomal order from 1ptel to 22qtel. Chromosomes are indicated with black and grey bars. Red, increases in DNA copy number; green, decreases in DNA copy number.

DNA copy number changes in a panel of 33 MFHs (Table 1) were analysed using a 1 Mb resolution BAC and PAC genomic microarray supplemented with the tiling-path between 1q12 and the beginning of 1q25. Hierarchical clustering of all samples based on the DNA copy number changes is shown in Figure 1. No associations between the clustering pattern and the clinical features were apparent. The panel consisted of mainly primary tumours and recurrences, and SAM was used to identify chromosomal regions with significantly different DNA copy number between the two subtypes, but no differences were found.

**Figure 2:**
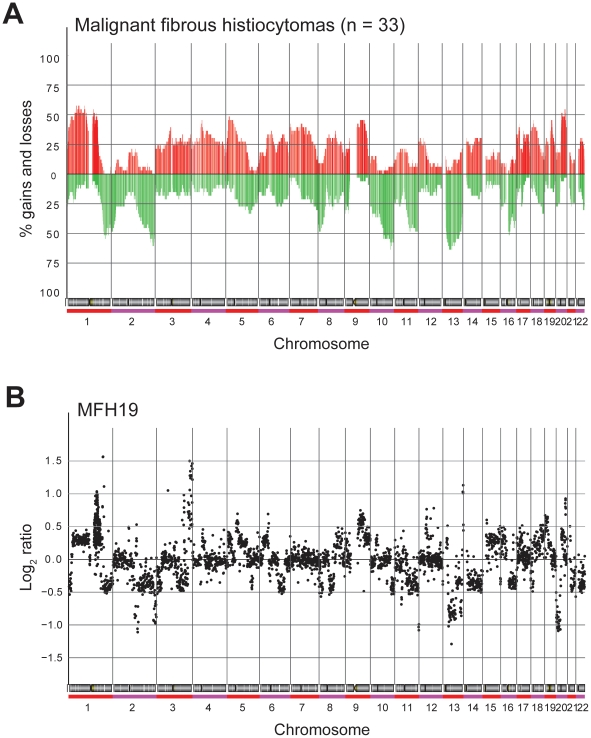
DNA copy number alterations. (**A**) Genome-wide frequency plot of copy number alterations identified by ACE in 33 MFHs. Red, increases in DNA copy number; green, decreases in DNA copy number. (**B**) Whole genome DNA copy number profile of a representative MFH. Log_2_ ratio for each of the genomic clones is plotted according to chromosome position.

Regions with significant DNA copy number changes were identified in each sample using the ACE algorithm in CGH-Explorer. The resulting frequency plot of gains and losses is shown in Figure 2A, and a representative genome-wide ratio plot for this type of tumours is shown in Figure 2B. The ratio plots for all samples are shown in Figure S1. Minimal recurrent regions of alteration identified by ACE in at least 10/33 (>30%) samples are presented in Table 2. The complete list of data of all defined regions of gain and loss from the ACE analysis is presented in Table S1.

**Table 2:** Minimal recurrent regions altered in malignant fibrous histiocytomas (n = 33).

Cytoband	Aberration	Start clone	End clone	Size (Mb)	Frequency
1p33-p32.3	Gain	RP1-86A18	RP4-631H13	2.6	19/33
1p31.3-p31.2	Gain	RP11-5P4	RP5-1033K19	5.5	19/33
1p21.3	Gain	RP11-17C2	RP11-413P11	1.2	19/33
1q21.2-q21.3	Gain	RP11-363I22	RP11-316M1	0.5	18/33
1q41	Loss	RP11-323K10	RP11-241C9	2.3	18/33
2p25.3-p25.1	Loss	RP11-352J11	RP11-542B5	6.0	16/33
2q36.3-q37.2	Loss	RP11-70L16	RP11-84G18	6.9	20/33
3p12.1-p11.2	Gain	RP11-382L10	RP11-312H1	3.9	13/33
4q12	Gain	RP11-738E22	RP11-355L4	0.2	15/33
5p14.3	Gain	RP11-28P24	RP11-26L18	2.8	16/33
5q23.2-q31.3	Loss	CTB-54G2	RP11-515C16	13.2	11/33
6q12-q13	Gain	RP1-160B9	RP11-256L9	3.3	12/33
7p22.1	Gain	RP11-172O13	RP1-42M2	0.4	14/33
7q11.21-q11.22	Gain	RP11-340I6	RP11-471N21	8.5	14/33
7q36.1-q36.3	Loss	RP11-24N19	CTB-3K23	10.4	10/33
8p23.2-p22	Loss	RP11-245H16	RP11-44L18	9.9	16/33
8q22.1	Gain	RP11-266D22	RP11-3D19	2.6	12/33
9p24.2-p24.1	Loss	RP11-509J21	RP11-509D8	1.5	11/33
9q21.33-q31.3	Gain	RP11-280P22	RP11-470J20	24.1	15/33
10q25.3-q26.11	Loss	RP11-96N16	RP11-5G18	1.1	21/33
11q14.1	Loss	RP11-118L16	RP11-482L11	1.9	16/33
11q14.3-q21	Loss	RP11-268B20	RP11-83E23	2.2	16/33
11q23.3-q25	Loss	CTD-3245B9	RP11-469N6	16.0	16/33
12p13.31-p13.1	Gain	RP11-277E18	RP11-377D9	5.2	10/33
13q13.3-q14.2	Loss	RP11-131F1	RP11-174I10	9.5	21/33
13q14.3-q21.1	Loss	RP11-384G23	RP11-516G5	3.0	21/33
14q23.3-q31.3	Gain	RP11-125H8	RP11-203D9	17.3	10/33
14q32.13-q32.33	Gain	RP11-371E8	CTC-820M16	13.7	10/33
16p13.2	Loss	RP11-114I12	RP11-148F10	1.3	10/33
16q12.1-q12.2	Loss	RP11-305A7	RP11-467J12	3.5	17/33
17p12	Gain	RP11-471L13	RP11-488L1	1.1	13/33
17p12-p11.2	Gain	RP11-459E6	RP11-404D6	0.6	13/33
17p11.2	Gain	RP11-189D22	RP11-78O7	1.8	13/33
17q21.31-q21.32	Gain	RP5-843B9	RP11-510P20	0.9	12/33
18q11.2	Gain	RP11-296E23	RP11-17J14	2.1	14/33
18q22.1-q23	Loss	RP11-21L20	RP11-118I2	9.0	11/33
19q13.11-q13.2	Gain	RP11-413M10	CTB-186G2	4.7	15/33
20q13.2	Gain	RP5-994O24	RP11-6L15	3.1	18/33
22q12.2-q12.3	Gain	RP1-76B20	CTA-415G2	3.4	10/33
22q12.3-q13.1	Gain	LL22NC01-132D12	CTA-228A9	1.4	10/33
22q13.2-q13.33	Loss	RP3-388M5	CTA-722E9	5.8	10/33

In general, the samples showed numerous gains and losses of large chromosomal regions. Of the 41 minimal recurrent regions identified in the tumour samples, 24 represented gains and 17 losses of chromosomal segments. In at least 30% of the tumours, gain of regions in chromosome 1, 3–9, 12, 14, 17–20 and 22 was identified (Table 2). The most frequent gains observed were in chromosome 1, where minimal recurrent regions in 1p33-p32.3 (2.6 Mb), 1p31.3-p31.2 (5.5 Mb) and 1p21.3 (1.2 Mb) were gained in 19/33 (58%) samples, whereas 1q21.2-q21.3 (0.5 Mb) was gained in 18/33 (55%) samples. Gain of 20q13.2 (3.1 Mb) was identified in 18/33 (55%) samples. Scattered high-level amplification (log_2_ ratio>1) was observed in some of the tumours, but not consistently in any region (see Table S1).

Loss of regions in chromosome 1, 2, 5, 7–11, 13, 16, 18 and 22 was identified in at least 30% of the tumours (Table 2). The most frequent losses observed were in chromosome 10 and 13, where minimal recurrent regions in 10q25.3-q26.11 (1.1 Mb), 13q13.3-q14.2 (9.5 Mb) and 13q14.3-q21.1 (3.0 Mb) were lost in 21/33 (64%) samples. Loss of 2q36.3-q37.2 (6.9 Mb) was identified in 20/33 (61%) samples, whereas loss of 1q41 (2.3 Mb) was identified in 18/33 (55%) samples and 16q12.1-q12.2 (3.5 Mb) in 17/33 (52%) samples. Scattered homozygous deletion (log_2_ ratio <−1) was observed in some of the tumours, but not consistently in any region (see Table S1).

**Figure 3:**
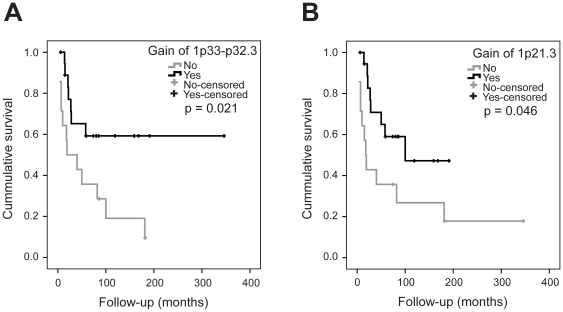
Patient survival curves. Kaplan-Meier plots with overall survival curves for (A) patients with gain of 1p33-p32.3 (n = 19) and patients with normal copy number or loss (n = 14) and (B) patients with gain of 1p21.3 (n = 19) and patients with normal copy number or loss (n = 14).

Clinical correlatesStatistical analyses were performed in order to identify possible correlations between the clinical information (Table 1) and the minimal recurrent chromosomal regions altered (Table 2). Survival analysis revealed that gain of 1p33-p32.3 and 1p21.3 was significantly associated with better patient survival (*P* = 0.021 and 0.046, respectively). Figure 3A and -B shows the corresponding Kaplan-Meier plots with overall survival curves. In contrast, male gender and metastasis at diagnosis were significantly associated with poor patient survival (*P* = 0.019 and 0.006, respectively). The corresponding Kaplan-Meier plots are shown in Figure S2.

Multivariate analysis was performed in order to identify the most important prognostic factors. All experimental and clinical variables that were significantly associated with poor survival were tested using Cox regression. None of the variables were identified as independent prognostic factors, but metastasis at diagnosis showed almost significance as an independent prognostic factor (relative risk 4.0, *P* = 0.059).

### Comparison with leiomyosarcomas

**Figure 4:**
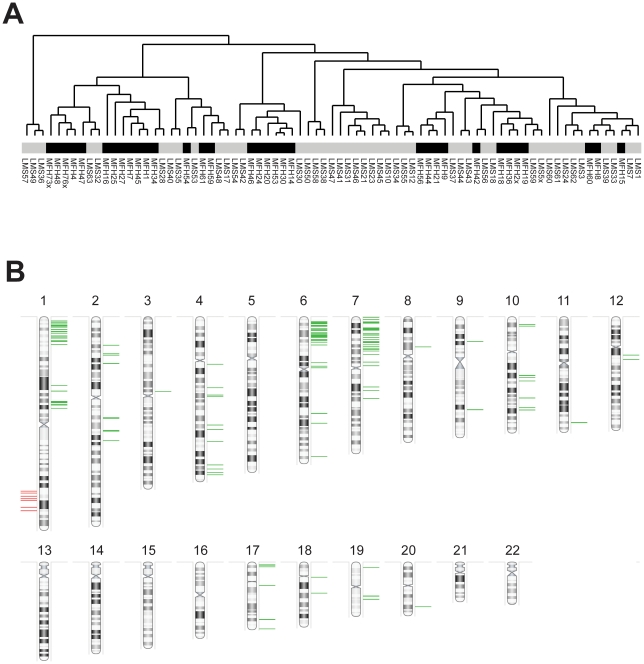
Comparison between MFHs and leiomyosarcomas. (**A**) Hierarchical clustering dendogram of 33 MFHs and 44 leiomyosarcomas using DNA copy number ratios relative to a pool of normal diploid DNA. Black, MFHs; grey, leiomyosarcomas. (**B**) Graphical representation of genomic clones identified by SAM to be significantly different between the MFHs and leiomyosarcomas. The lines represent the chromosomal position of the 156 identified genomic clones. Green, higher copy number in MFHs compared to leiomyosarcomas; red, lower copy number.

In order to investigate differences in DNA copy number aberrations between MFHs and leiomyosarcomas, a comparison with similar array CGH data from a panel of 44 leiomyosarcomas ([12] and Kresse et al., unpublished) was done. Figure 4A shows the hierarchical clustering dendogram of the 33 MFHs and 44 leiomyosarcomas. Although smaller groups of MFHs and leiomyosarcomas clustered separately, there were no overall significant differences in the clustering pattern between the two tumour types.

SAM was used to identify chromosomal regions with significantly different DNA copy number between the MFHs and leiomyosarcomas. Using a false discovery rate of <1%, 156 genomic clones showing significant copy number differences between the two tumour types were identified. Figure 4B shows a graphical representation of the genomic clones, and the complete list is given in Table S2. Seven chromosomal segments represented by multiple significant clones were identified; 1p36.32-p35.2, 1p21.3-p21.1, 1q32.1-q42.13, 2q14.1-q22.2, 4q33-q34.3, 6p25.1-p21.32 and 7p22.3-p13. Of the 156 identified genomic clones, 104 mapped to these seven regions. The percentage of genomic clones in the identified regions showing significant differences varied between 25–100%, with the 1p21.3-p21.1 region showing the highest percentage of clones with significant differences (see Table S2). The 1q32.1-q42.13 region showed significantly lower copy number in MFHs compared to leiomyosarcomas, whereas all the other six regions showed significantly higher copy number.

## Discussion

We have used array CGH to analyse DNA copy number changes in a panel of 33 MFHs, in order to identify recurrent copy number alterations at high-resolution and thus identify loci that may contain novel candidate oncogenes and/or tumour suppressor genes. The tumours were pathologically revised at the time of the study and classified according to the latest WHO classification [1]. The panel consisted mainly of the most common MFH subtype, spindle cell/pleomorphic MFHs, as well as some myxofibrosarcomas and pleomorphic MFHs with giant cells (Table 1). Hierarchical clustering of the samples based on the DNA copy number changes showed no associations between the clustering pattern and the tumour subtype or the other clinical features (Figure 1), and no chromosomal regions with significantly different DNA copy number between the primary tumours and recurrences were found.

The samples showed numerous gains and losses of large chromosomal regions. Forty-one minimal recurrent regions were identified as altered in at least 30% of the tumour samples, of which 24 showed increased and 17 decreased copy number (Table 2). The most common gains observed were in chromosome 1, where minimal recurrent regions in 1p33-p32.3, 1p31.3-p31.2 and 1p21.3 were gained in 58% of the samples, whereas 1q21.2-q21.3 was gained in 55% of the samples. Increased copy number of regions in chromosome 1 has been frequently observed in MFHs previously, in particular 1p31 and 1q21-q22 [5], [6], [8], [9]. High-level amplification of the 1q21-q22 region has been frequently found [6], and this is also a common finding in other types of sarcomas, like osteosarcomas [17], [18].

Although, gain of the 1p31 region has previously been associated with a trend to decreased overall patient survival in MFHs [5], no such association was seen in this tumour panel. On the contrary, gain of 1p33-p32.3 and 1p21.3 were significantly associated with better patient survival (*P* = 0.021 and 0.046, respectively) (Figure 3A and –B). However, these aberrations were not identified as independent prognostic factors in the multivariate analysis including all experimental and clinical variables significantly associated with survival. Notably, the other region in 1p frequently gained, 1p31.3-p31.2, was not associated with better (or worse) patient survival.

Gain of 20q13.2 was also identified in 55% of the samples, and increased copy number of regions in 20q has also been frequently observed in MFHs previously [4], [6], [8], [9]. Other regions of frequent gain including 5p14.3, 4q12, 9q21.33-q31.3 and 19q13.11-q13.2 (Table 2) have also been frequently observed in MFHs [4], [5], [6], [9], [10], [11], [19]. However, this higher resolution analysis also identified rather frequent regions of gain in MFHs (around 40%), not previously reported as such, including 7q11.21-q11.22, 18q11.2, 3p21.1-p11.2 and 6q12-q13.

The most frequent losses observed were in chromosome 10 and 13, where minimal recurrent regions in 10q25.3-q26.11, 13q13.3-q14.2 and 13q14.3-q21.1 were lost in 64% of the samples. Decreased copy number of regions in chromosome 13 has been frequently observed in MFHs previously [4], [5], [6], [8], [9], [10], [20]. The losses may involve the whole chromosome arm, or more specific regions like 13q14 and 13q21-q22. Loss of regions in chromosome 13 is a frequent finding in other types of sarcomas as well [7], [12], [21], [22], and the well-known tumour suppressor gene *RB1* is considered to be the prime candidate target for the deletion involving the 13q14 region. The *RB1* gene has also previously been suggested to be the target for loss of the 13q14-q21 region in MFHs [20].

Loss of regions of chromosome 10 is also a frequent finding in MFHs [4], [6], [8], as well as other types of sarcomas [12], [21], [22]. The minimal recurrent region identified here was 10q25.3-q26.11, and frequent loss of 10q25 has also been previously reported in MFHs [6]. Other recurrent regions of loss included 2q36.3-q37.2 (61% of the samples), 1q41 (55%), 16q12.1-q12.2 (52%), 2p25.3-p25.1, 11q14.1, 11q14.3-q21 and 11q23.3-q25 (all 48%), all previously reported as recurrent in MFHs [4], [6], [7], [8]. However, recurrent regions of loss not previously reported as frequent in MFHs were also identified, including 8p23.2-p22 (48% of the samples) and 5q23.2-q31.3 (33%).

Although several of the minimal recurrent regions identified were small in size, a number of genes are located in these segments, making it challenging to identify the target genes for the chromosomal aberrations based on these data only. Further analysis utilizing gene expression and functional data would be necessary for determining the most likely candidate target genes.

One of the main challenges in diagnosing MFHs is to distinguish them from other malignant tumours with a similar degree of cellular pleomorphism, like pleomorphic leiomyosarcomas, rhabdomyosarcomas and liposarcomas [3]. In order to investigate differences in DNA copy number aberrations between MFHs and leiomyosarcomas, a comparison with similar array CGH data from a panel of 44 leiomyosarcomas ([12] and Kresse et al., unpublished) was done. Hierarchical clustering of the samples based on the DNA copy number changes showed no major differences in the clustering pattern between the two tumour types (Figure 4A), similar to what has been reported by others [19], [23].

Seven chromosomal segments represented by multiple significant clones were identified as significantly different in copy number between the MFHs and leiomyosarcomas; 1p36.32-p35.2, 1p21.3-p21.1, 1q32.1-q42.13, 2q14.1-q22.2, 4q33-q34.3, 6p25.1-p21.32 and 7p22.3-p13 (Figure 4B). The 1q32.1-q42.13 region showed significantly lower copy number in MFHs compared to leiomyosarcomas, whereas all the other six regions showed significantly higher copy number. In a previous study comparing CGH data from 102 MFHs and 82 leiomyosarcomas, several chromosomal regions with differences in copy number between the two groups were identified [23]. Interestingly, the 1ptel-1p31 and 7p22-p15 regions were also shown to be more frequently gained in the MFHs, whereas the 1q32-qtel region was shown to be more frequently gained in the leiomyosarcomas, consistent with our findings. In addition, loss of the 6p region was more frequent in the leiomyosarcomas [23]. However, in another study comparing array CGH data from 31 MFHs/UPSs and 18 leiomyosarcomas, no chromosomal regions with significant differences in copy number were found using SAM, only a small set of single clones [19]. It is still uncertain whether MFHs represent a separate entity or if the majority correspond to highly pleomorphic leiomyosarcomas, but our work identified genomic differences between these two tumour groups and supports the existence of two separate entities.

Previously it was shown that a subset of MFHs could correspond to undifferentiated liposarcomas since these showed similar chromosomal aberrations, like high-level amplification of the 12q14-q15 region [24]. Further it was demonstrated that these MFH samples showed frequent co-amplification of either 1p32 or 6q23, which was not observed in the liposarcomas, suggesting that the lack of differentiation may be a consequence of amplification of target genes located in these regions, like the *ASK1* (*MAP3K5*) gene in 6q23 [24], [25]. Increased copy number of regions in 12q was observed in this tumour panel as well, but not as frequent. Only two samples showed high-level amplification of parts of the region, but co-amplification of 1p32 or 6q23 was not identified in these samples (see Table S1), suggesting that this is not a general feature.

In summary, our array CGH analysis of a panel of MFHs identified a number of recurrent regions of gain and loss, some of which were associated with clinical features. Several of the regions have also been identified as frequently altered in previous CGH studies of MFHs, and may be characteristic for this type of tumours, although not necessarily specific. A comparison with a panel of leiomyosarcomas showed that the two tumour types could not be distinguished based on the overall DNA copy number profiles, but several specific chromosomal regions with significant differences in copy number were identified. If consistently found in larger panels of tumours, these aberrations may be used as additional tools for the differential diagnosis of MFHs and leiomyosarcomas.

## Supporting Information

Figure S1 Genome-wide ratio plots of 33 MFHs. (PDF)

Figure S2 Kaplan-Meier plots with overall survival curves for A) female patients (n = 14) and male patients (n = 19) and B) patients with metastasis at diagnosis (n = 4) and patients without (n = 29). (TIF)

Table S1 Identification of minimal recurrent regions in malignant fibrous histiocytomas using ACE (FDR = 0.0001 and medium sensitivity). Orange areas indicate increased DNA copy number detected by ACE and green areas decreased DNA copy number; Bold numbers indicate high-level amplification (log2>1) or homozygous deletion (<−1); Red triangles indicate missing values imputed via a K-Nearest Neighbor algorithm normalization using SAM; Recurrent regions are defined in light grey (≥30%) and grey (≥50%) and minimal recurrent regions in black frames. (XLS)

Table S2 Identification of genomic clones significantly different in copy number between MFHs and leiomyosarcomas using SAM. Genomic areas significantly different are indicated in black frames. (XLS)
