# Supplementary material for: DNA Copy Number Changes in Human Malignant Fibrous Histiocytomas by Array Comparative Genomic Hybridisation
Source: PLoS One. 2010 Nov 9;5(11):e15378. doi: 10.1371/journal.pone.0015378 (PMC2976768; doi:10.1371/journal.pone.0015378)

MFH1

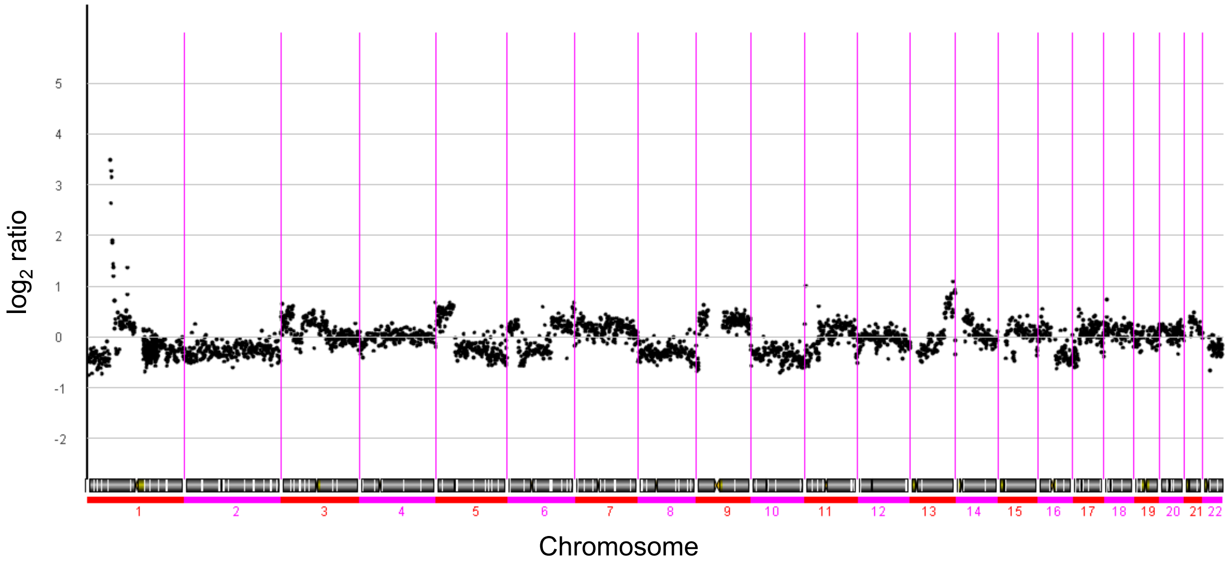

MFH2x

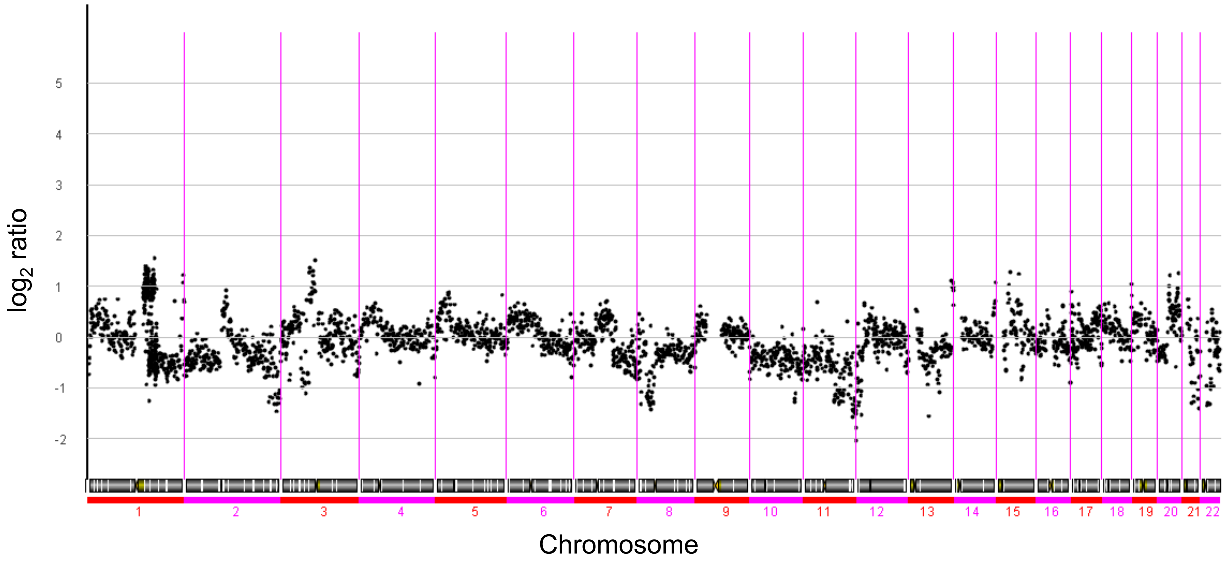

MFH4

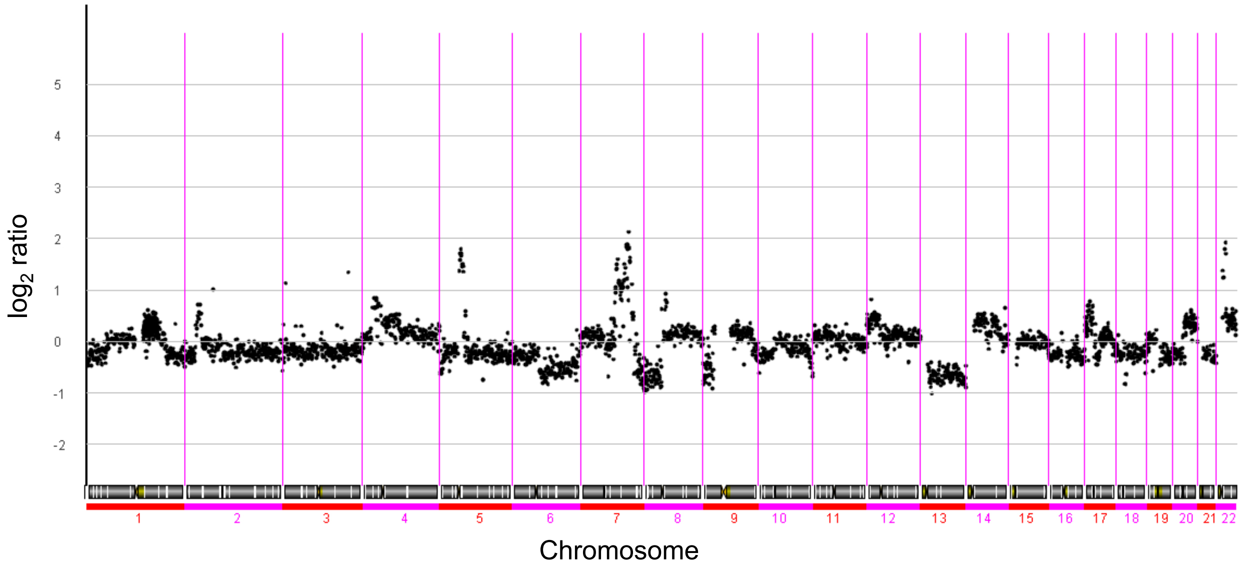

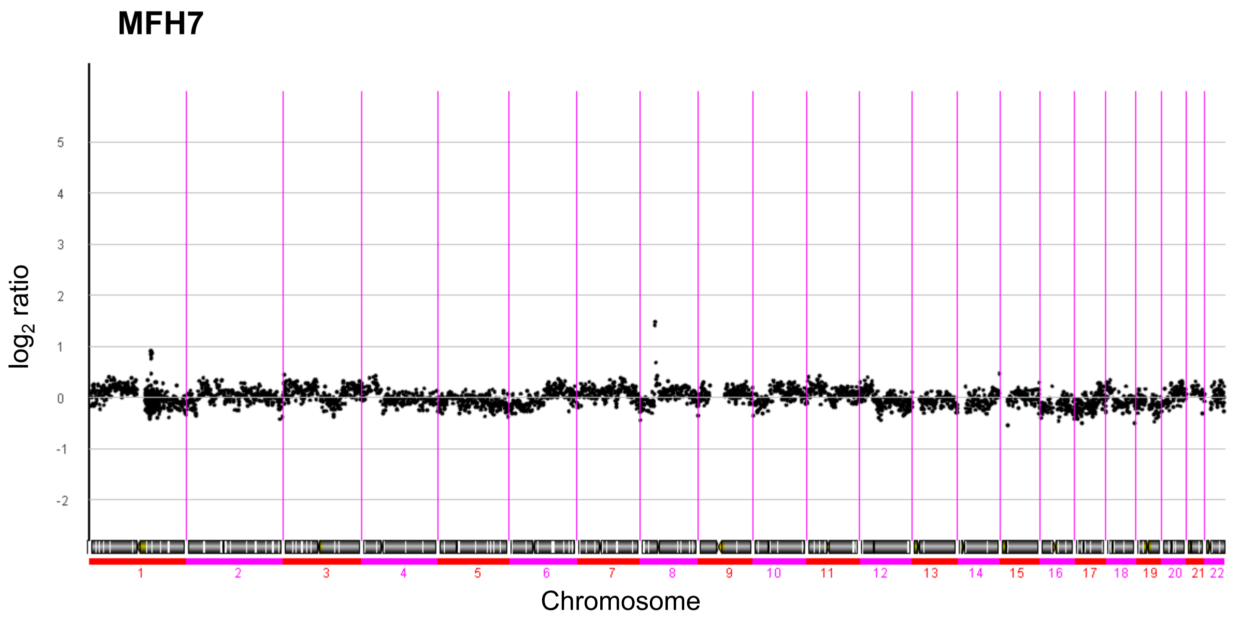
MFH7
log2 ratio
5
4
3
2
1
0
-1
-2
1 2 3 4 5 6 7 8 9 10 11 12 13 14 15 16 17 18 19 20 21 22
Chromosome

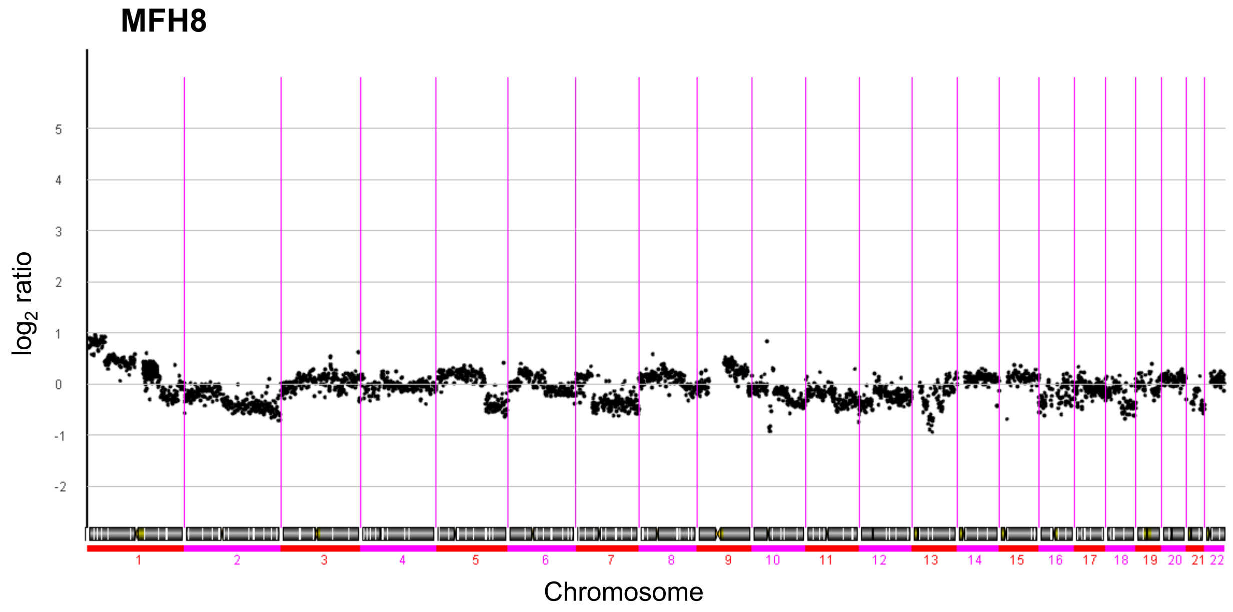
MFH8
log2 ratio
5
4
3
2
1
0
-1
-2
1 2 3 4 5 6 7 8 9 10 11 12 13 14 15 16 17 18 19 20 21 22
Chromosome

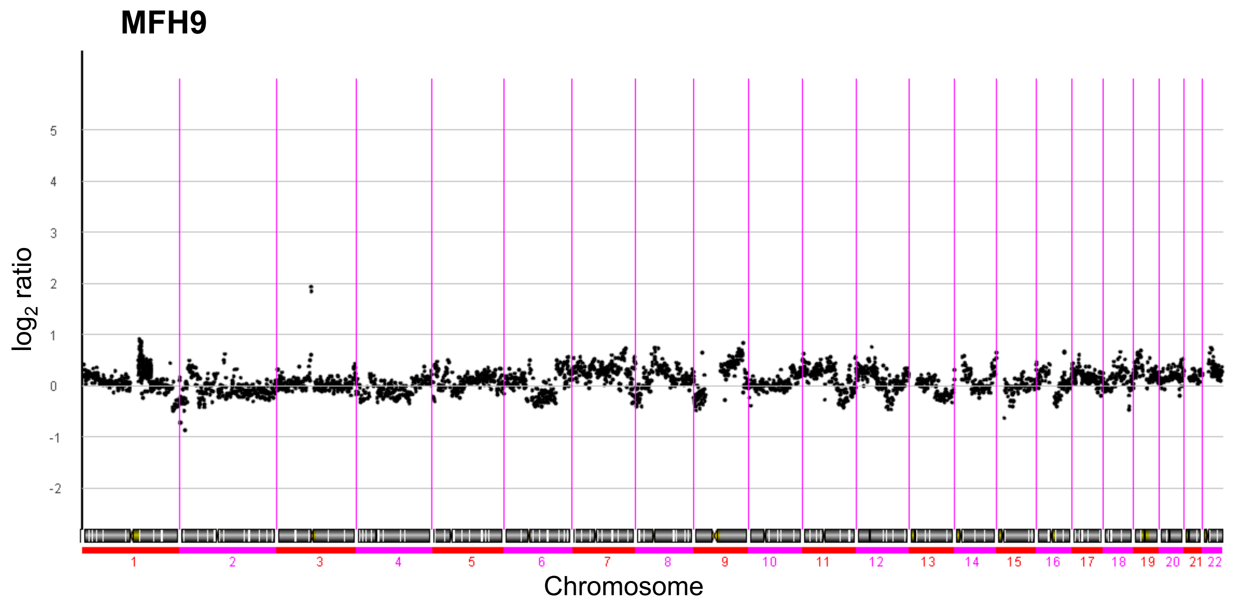
MFH9
log2 ratio
5
4
3
2
1
0
-1
-2
1 2 3 4 5 6 7 8 9 10 11 12 13 14 15 16 17 18 19 20 21 22
Chromosome

MFH14

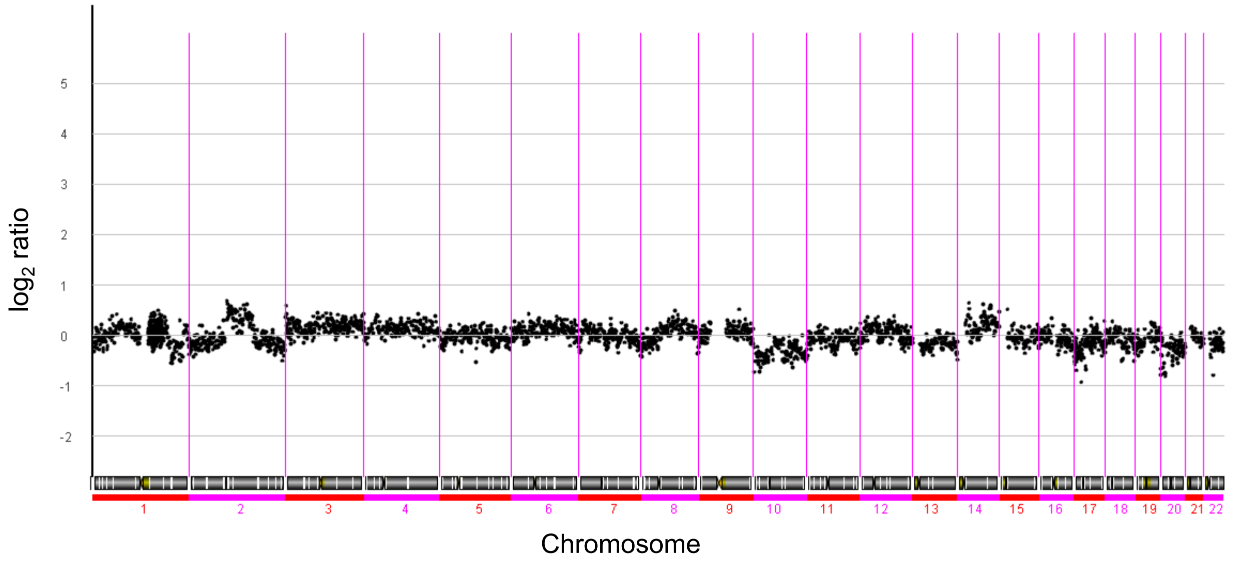

MFH15

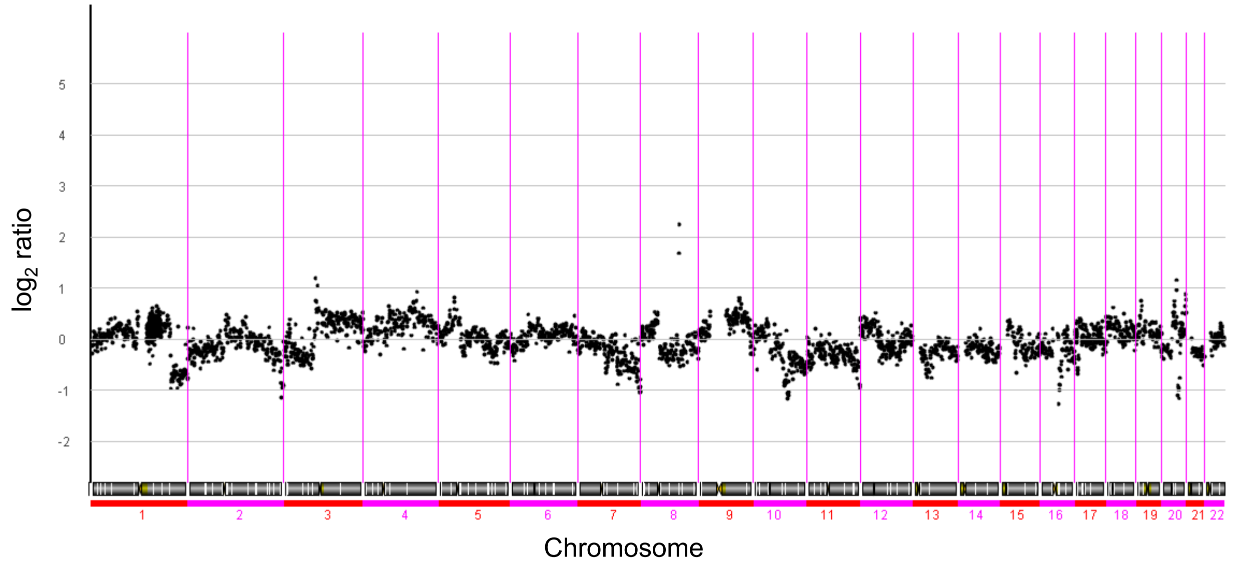

MFH16

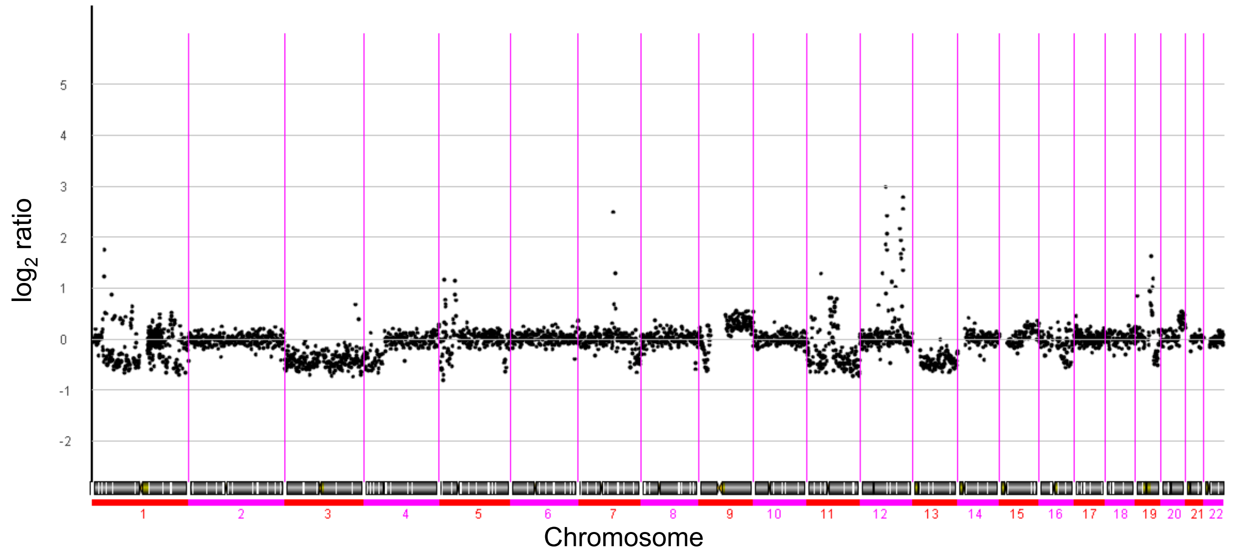

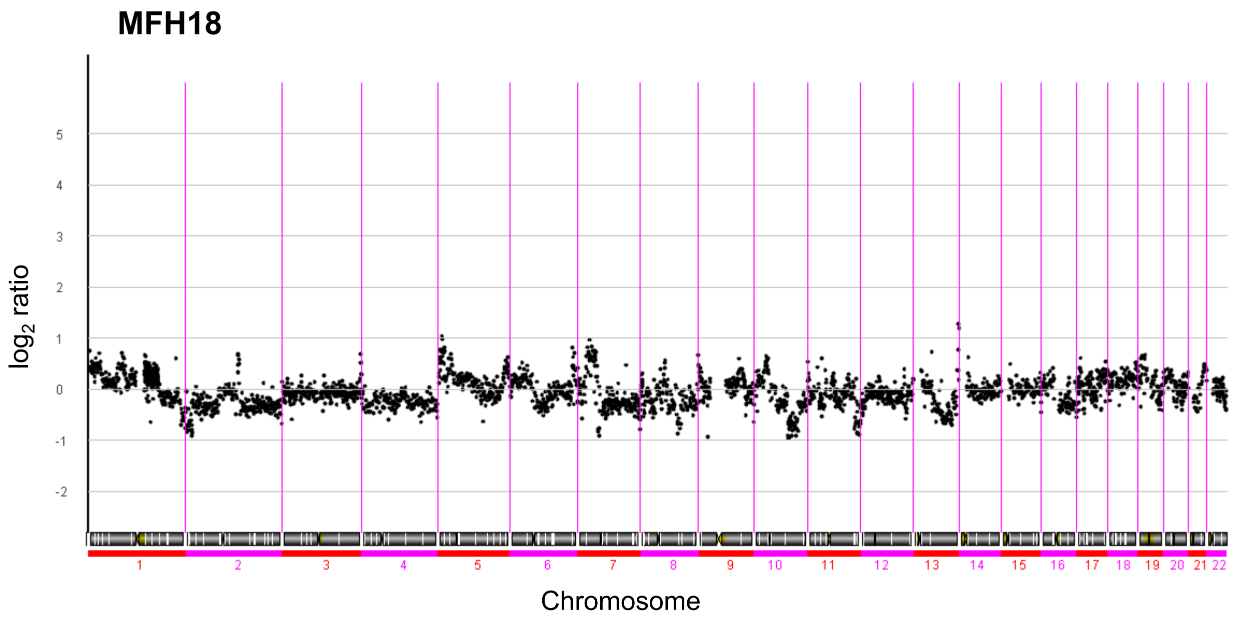
MFH18
log2 ratio
5
4
3
2
1
0
-1
-2
1 2 3 4 5 6 7 8 9 10 11 12 13 14 15 16 17 18 19 20 21 22
Chromosome

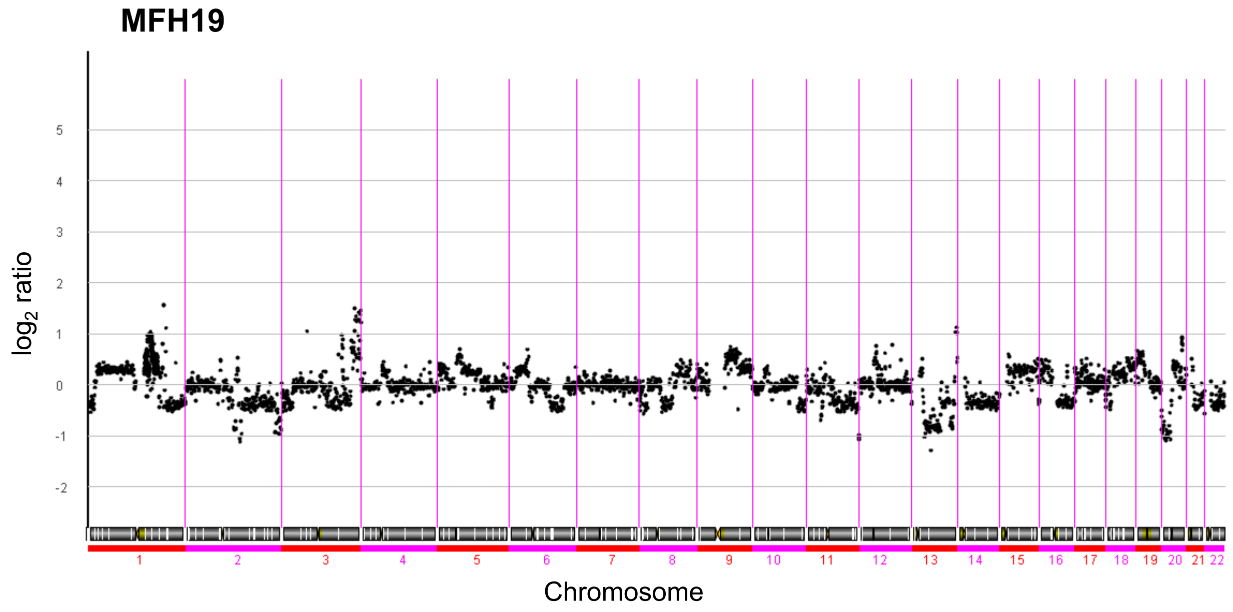
MFH19
log2 ratio
5
4
3
2
1
0
-1
-2
1 2 3 4 5 6 7 8 9 10 11 12 13 14 15 16 17 18 19 20 21 22
Chromosome

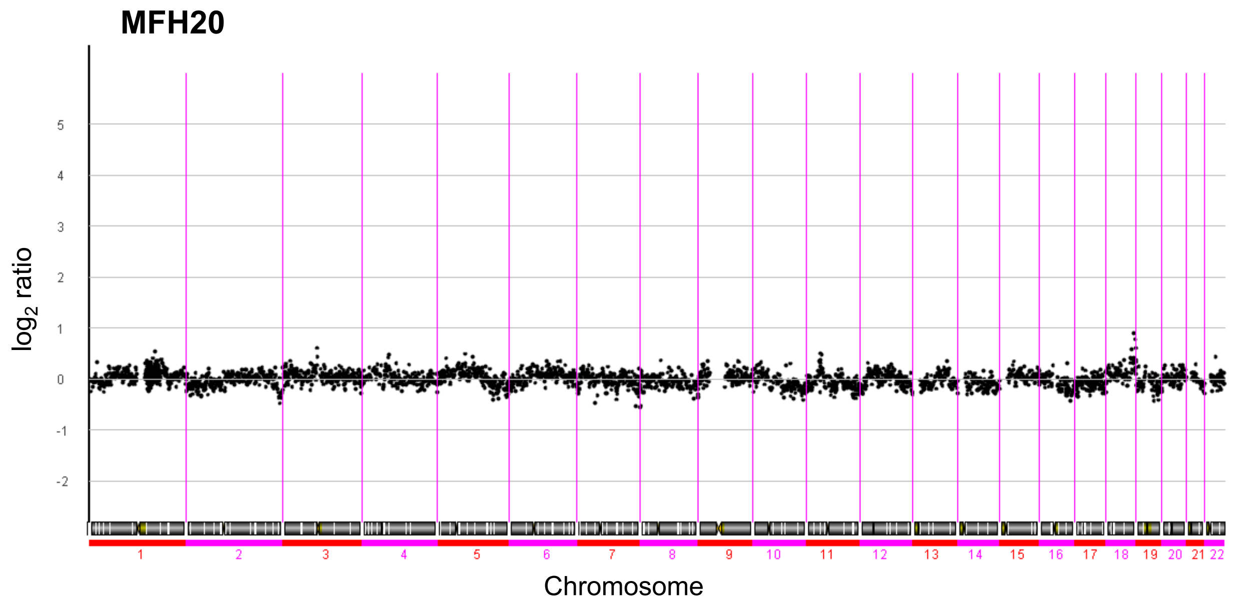
MFH20
log2 ratio
5
4
3
2
1
0
-1
-2
1 2 3 4 5 6 7 8 9 10 11 12 13 14 15 16 17 18 19 20 21 22
Chromosome

MFH21

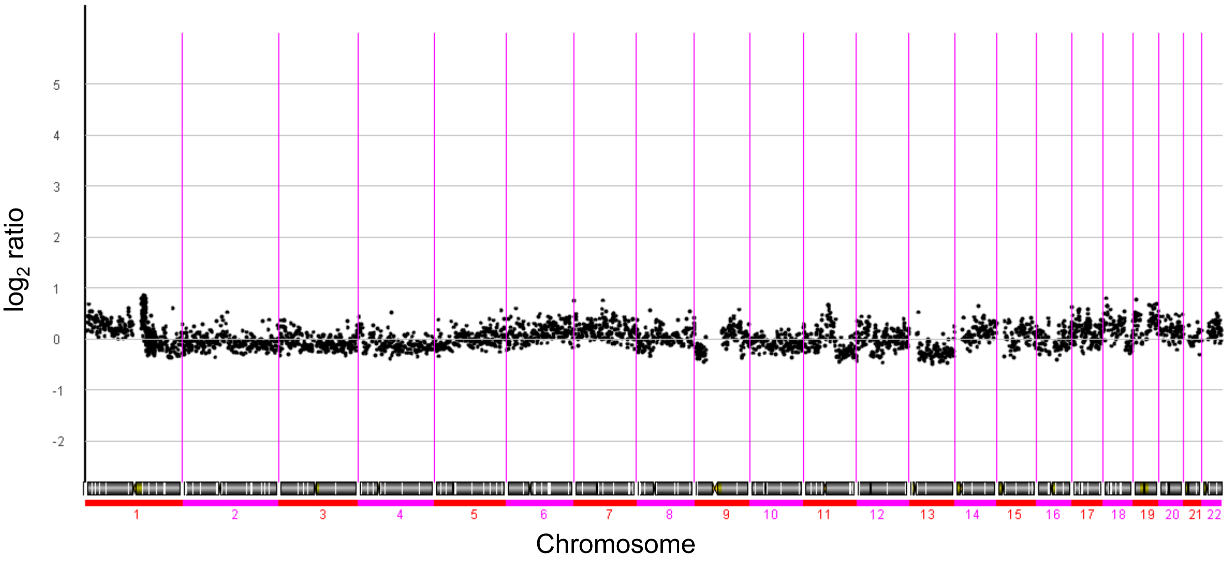

MFH24

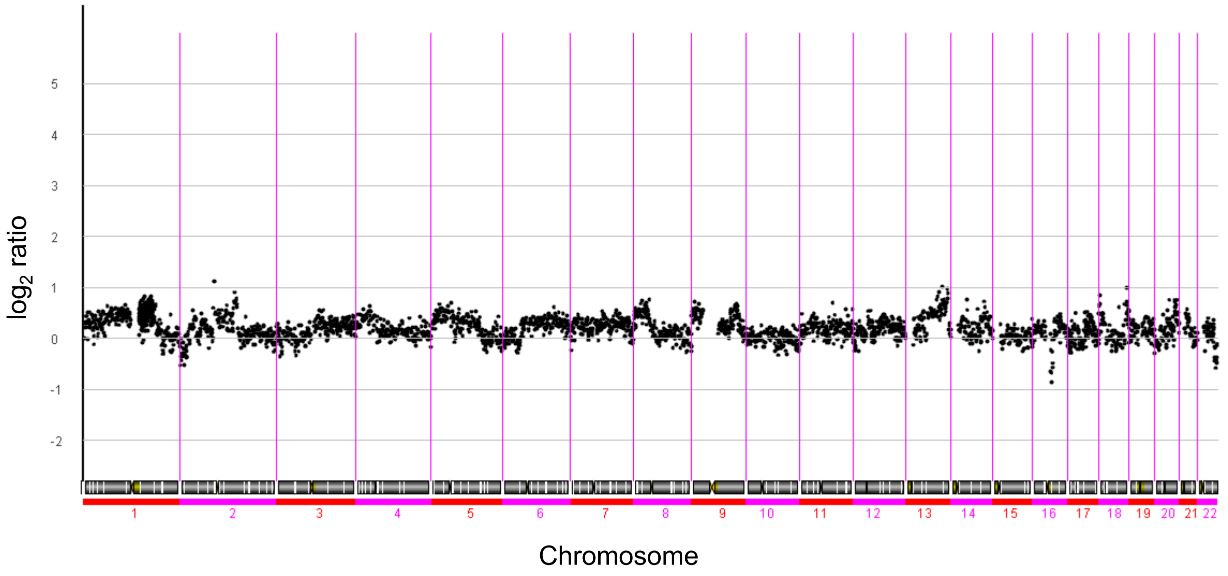

MFH25

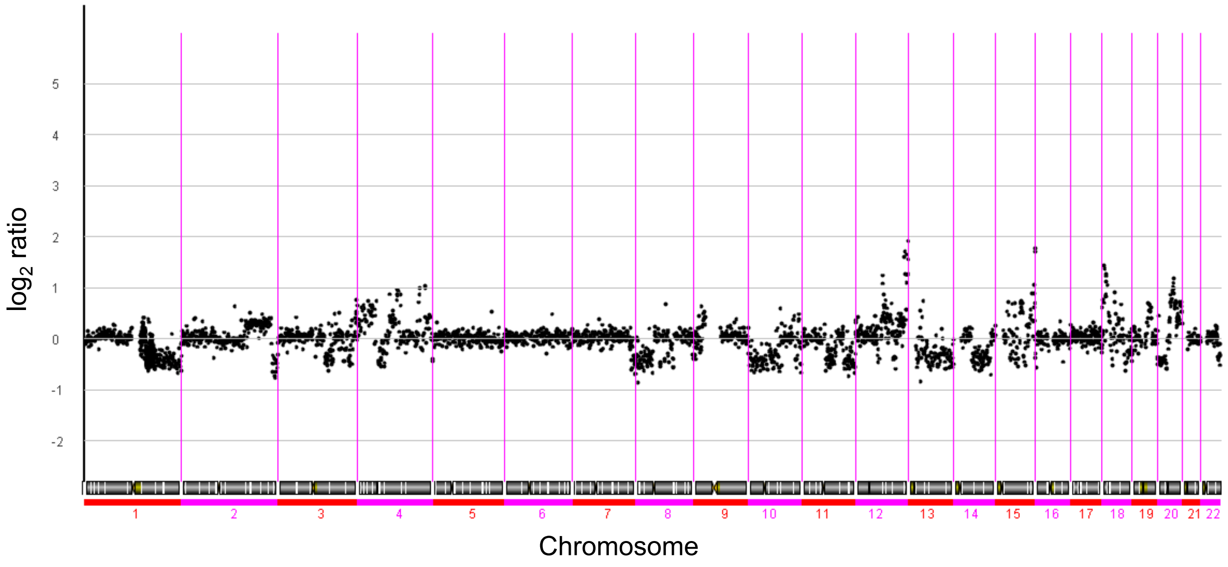

MFH27

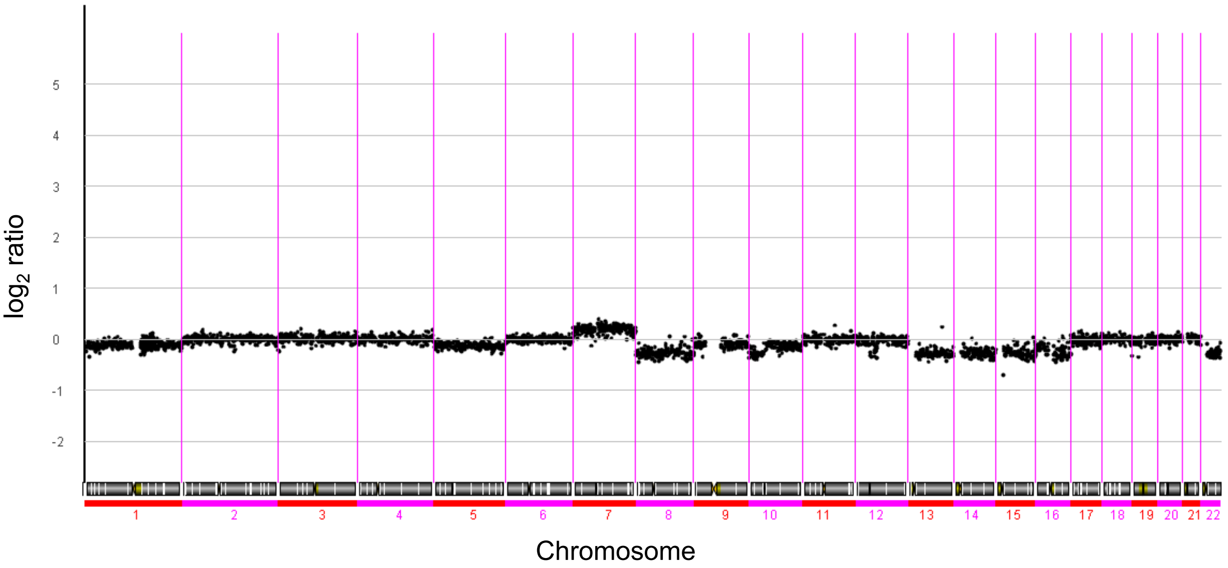

MFH30

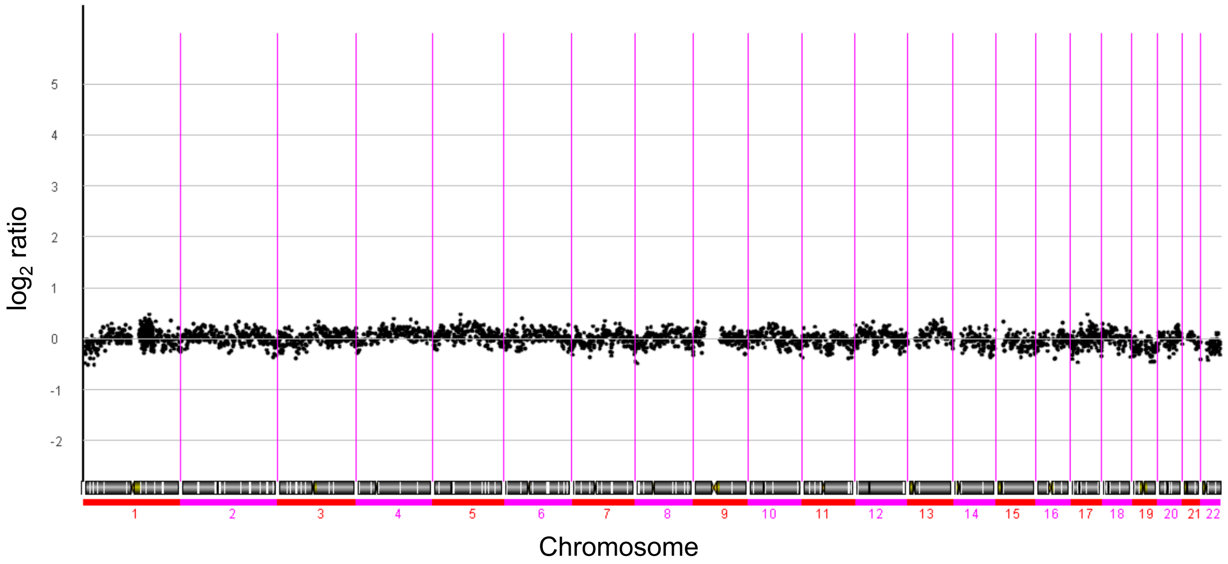

MFH34

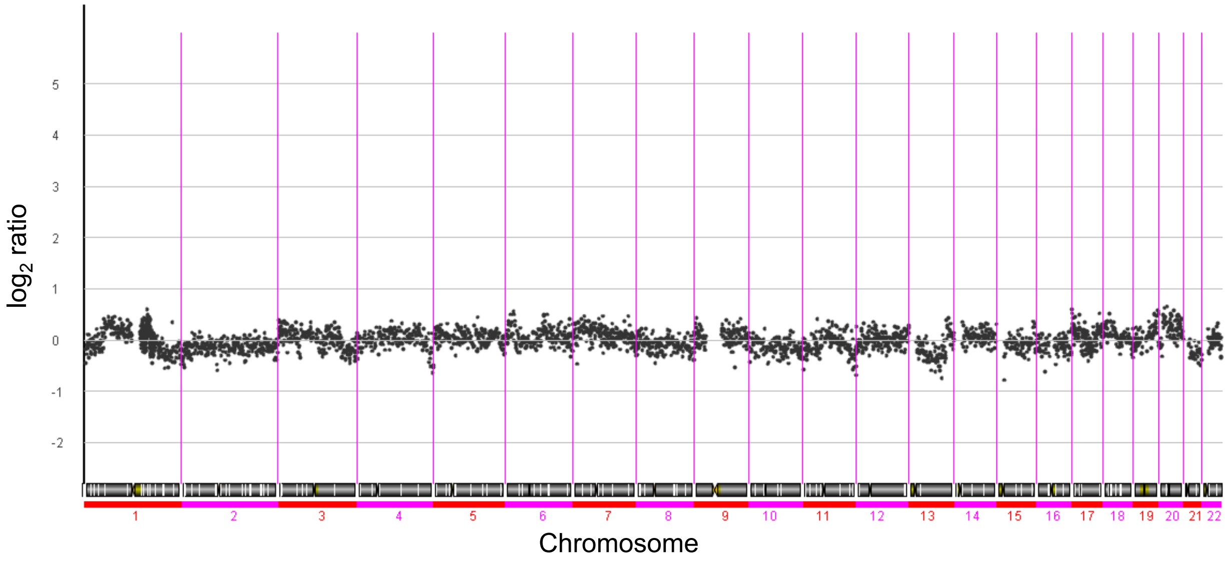

MFH36

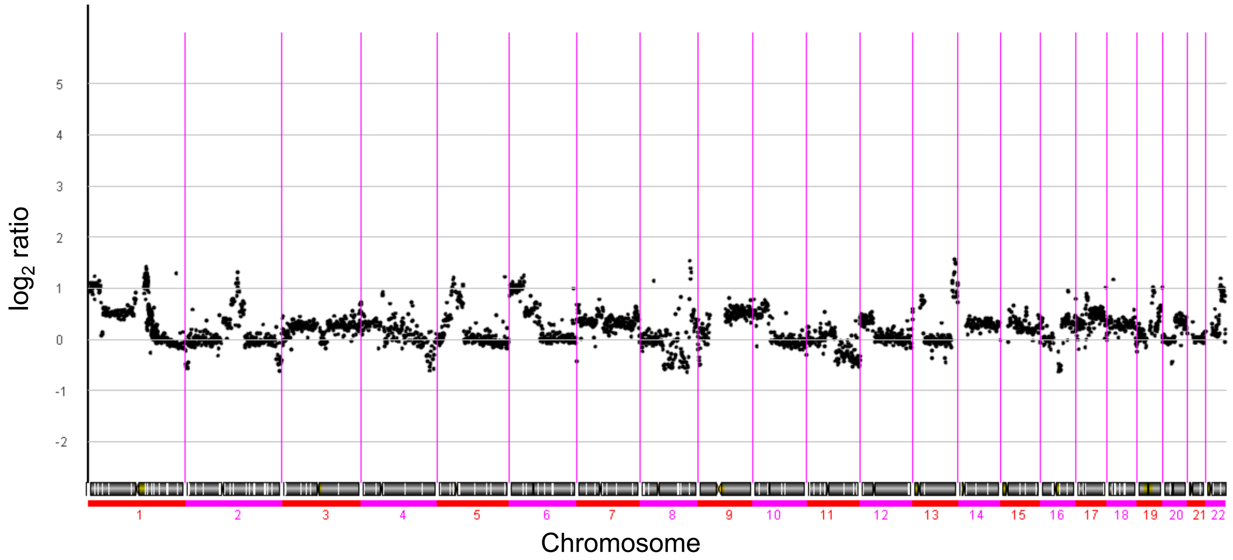

MFH42

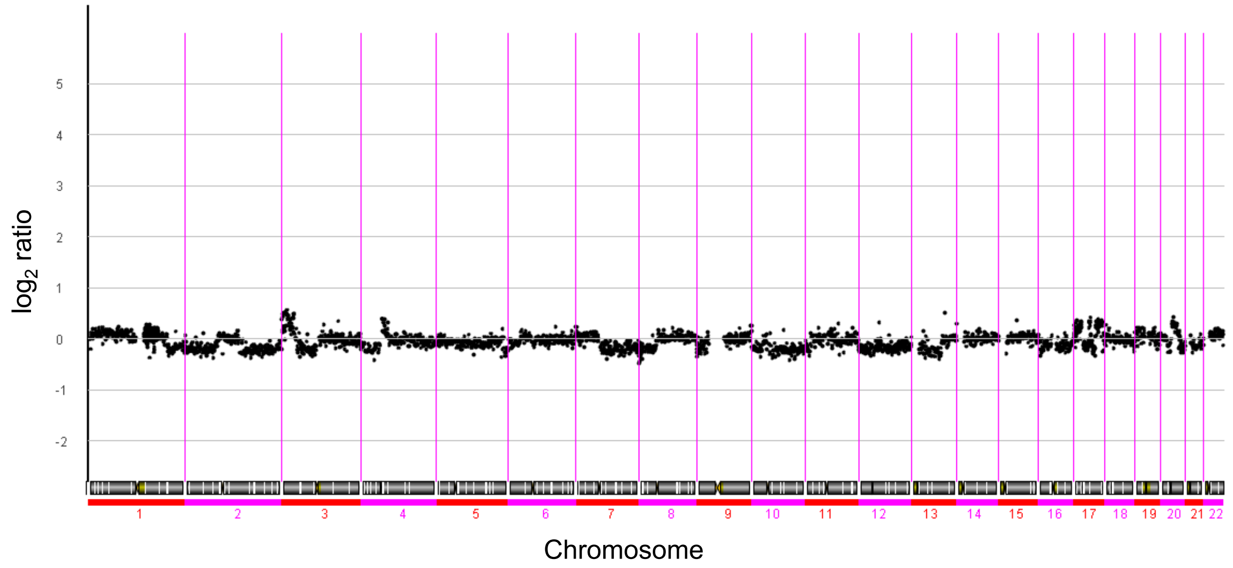

MFH44

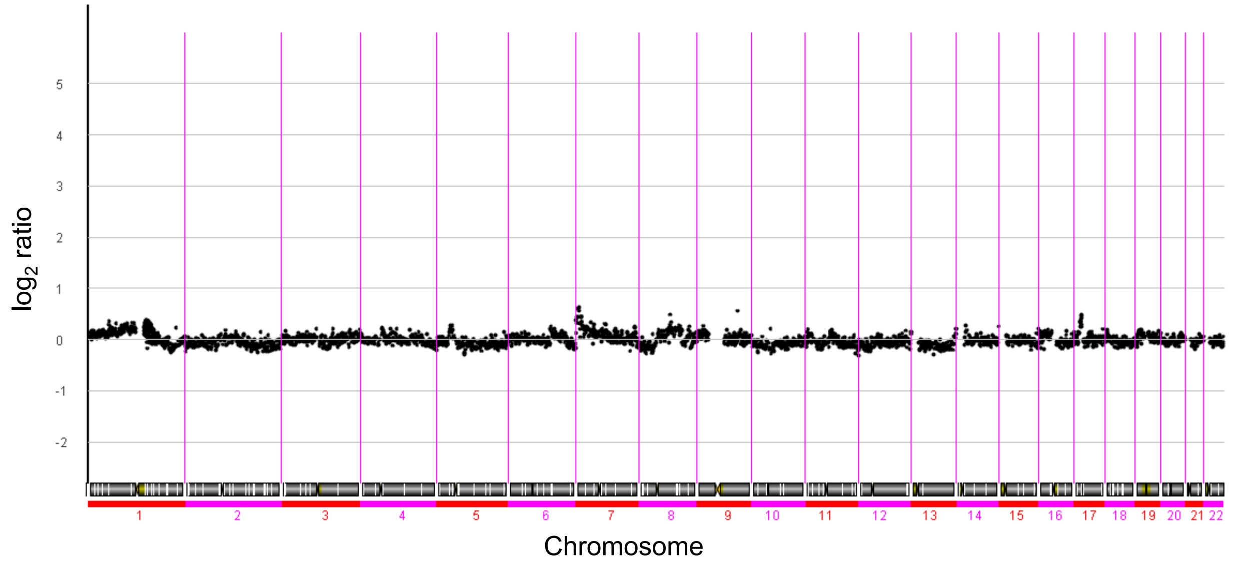

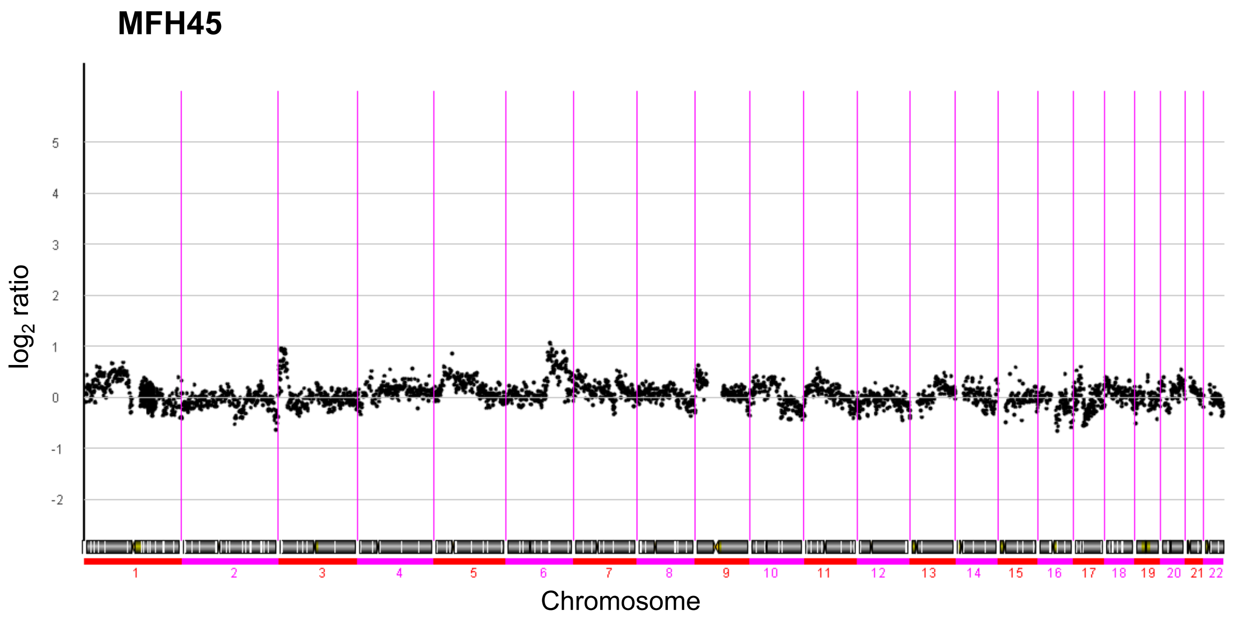
MFH45
log2 ratio
5
4
3
2
1
0
-1
-2
1
2
3
4
5
6
7
8
9
10
11
12
13
14
15
16
17
18
19
20
21
22
Chromosome

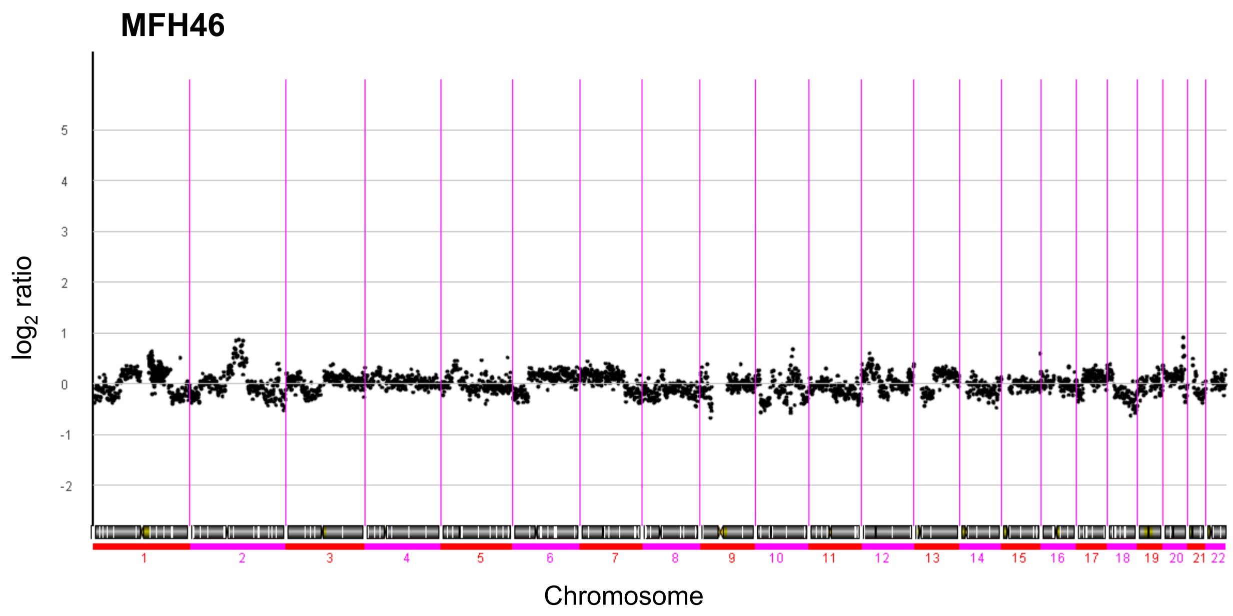
MFH46
log2 ratio
5
4
3
2
1
0
-1
-2
1
2
3
4
5
6
7
8
9
10
11
12
13
14
15
16
17
18
19
20
21
22
Chromosome

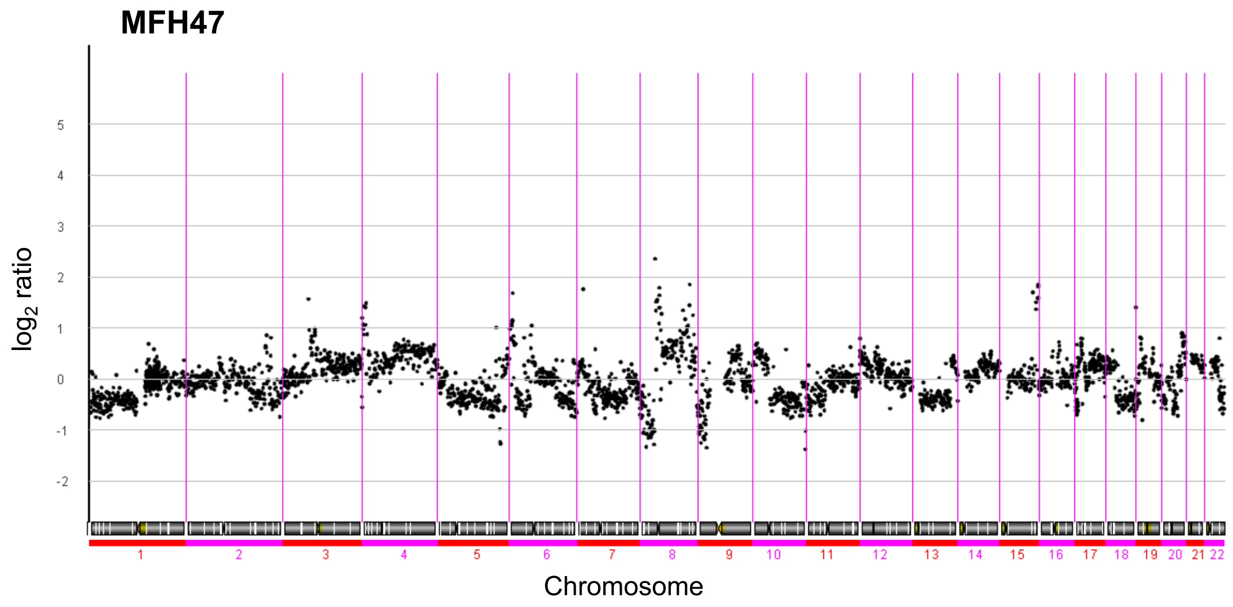
MFH47
log2 ratio
5
4
3
2
1
0
-1
-2
1
2
3
4
5
6
7
8
9
10
11
12
13
14
15
16
17
18
19
20
21
22
Chromosome

MFH48

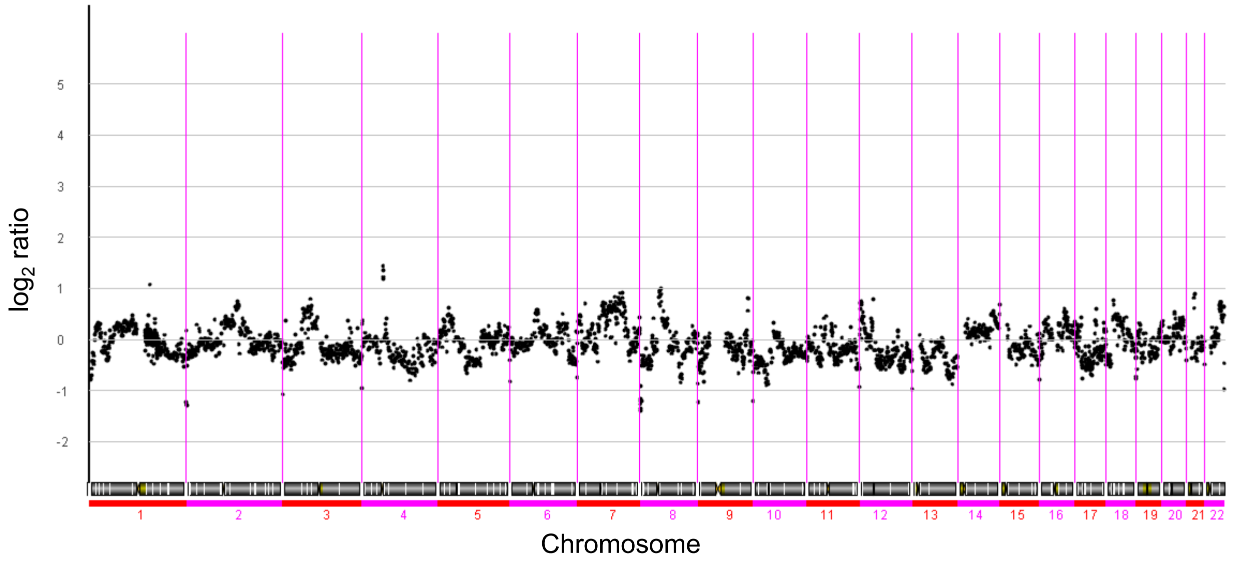

MFH53

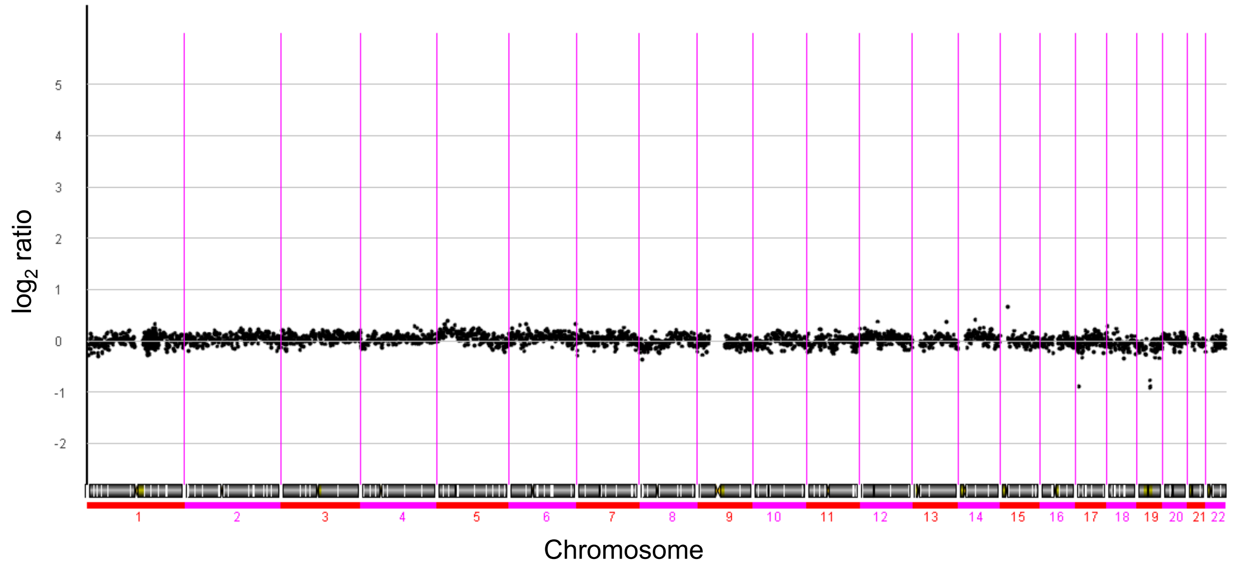

MFH54

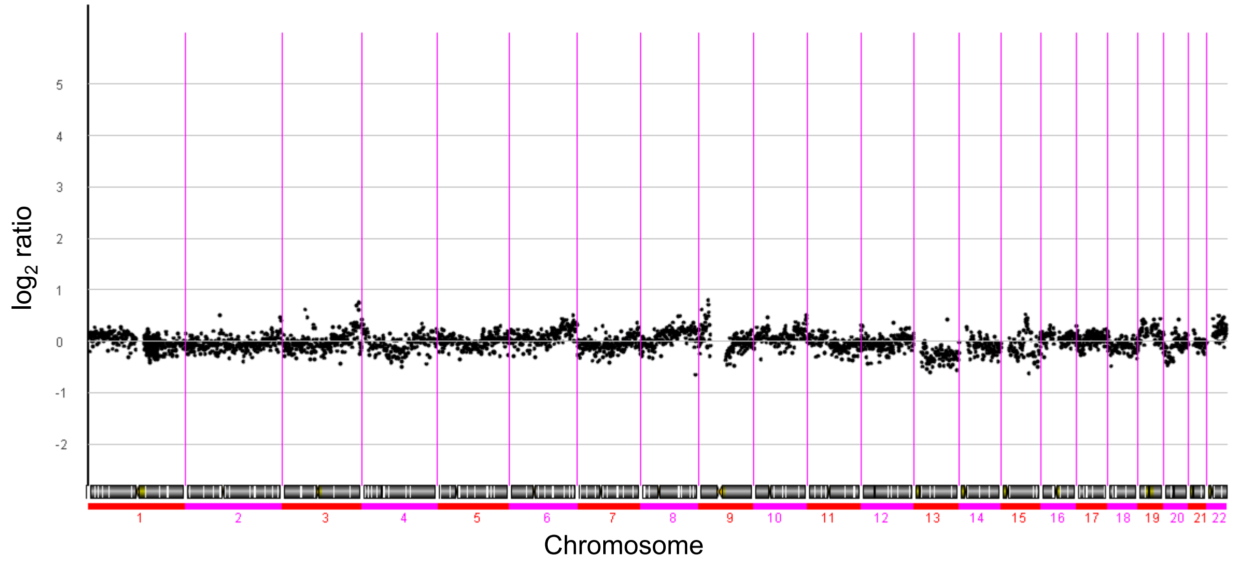

MFH56

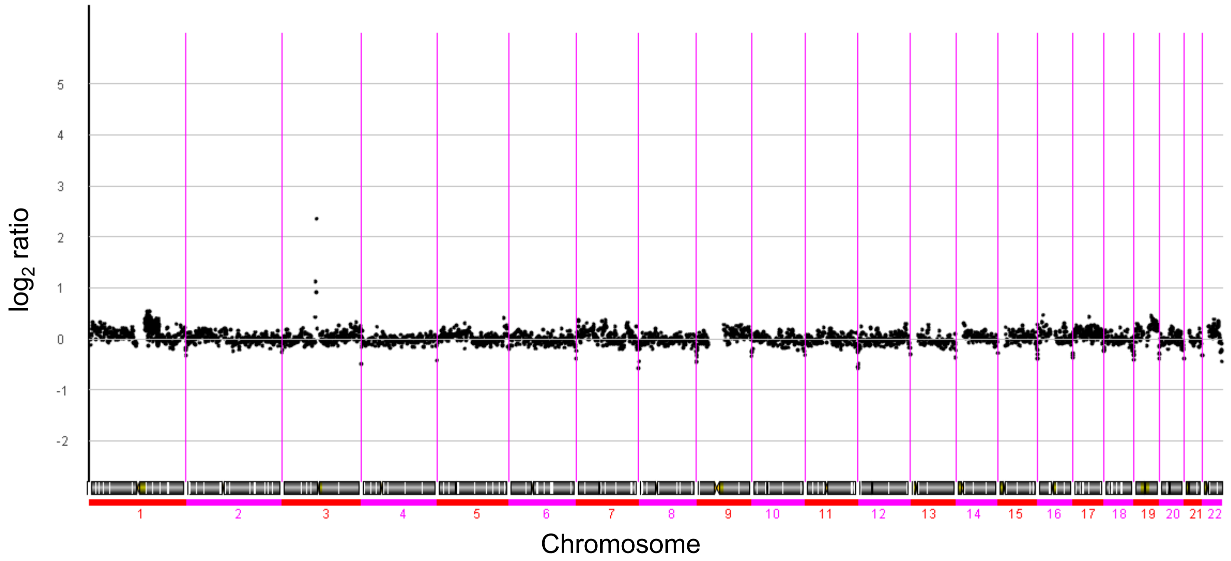

MFH59

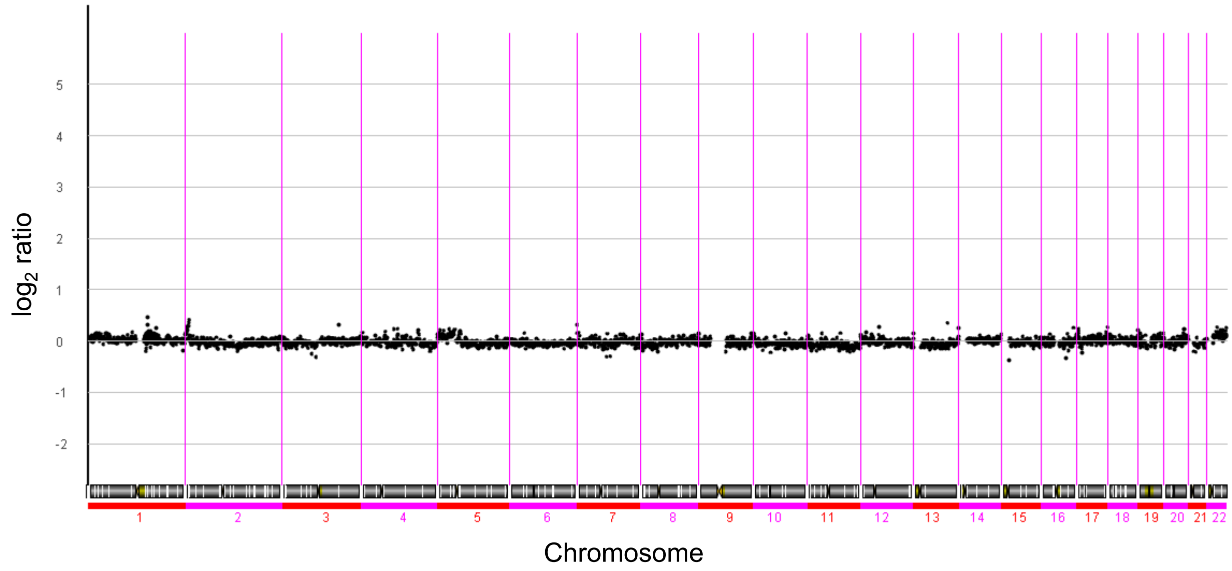

MFH60

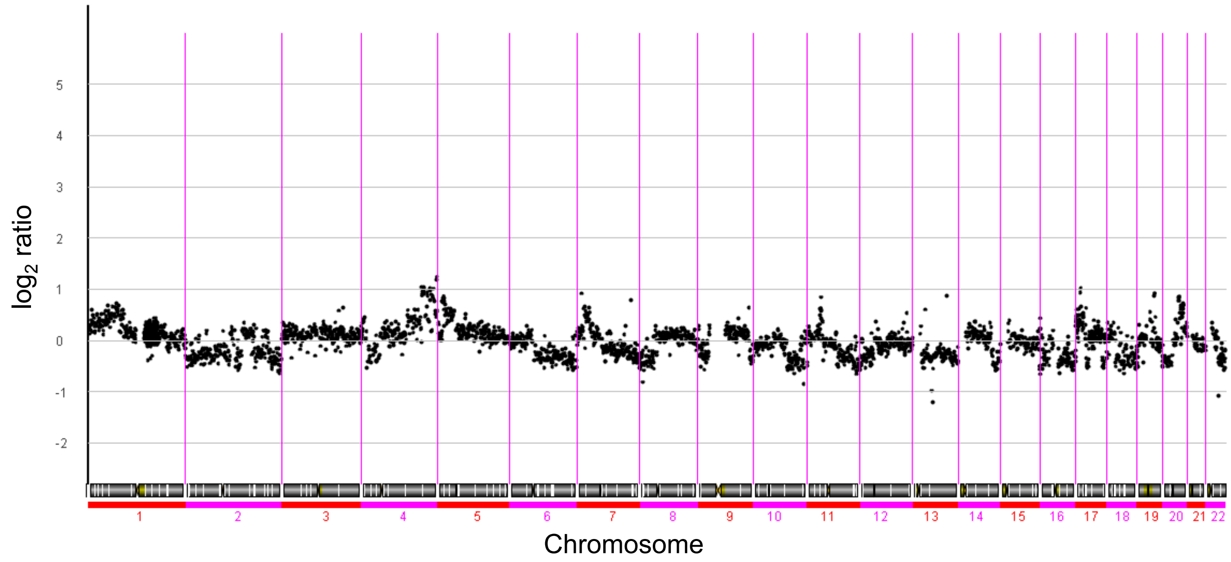

MFH61

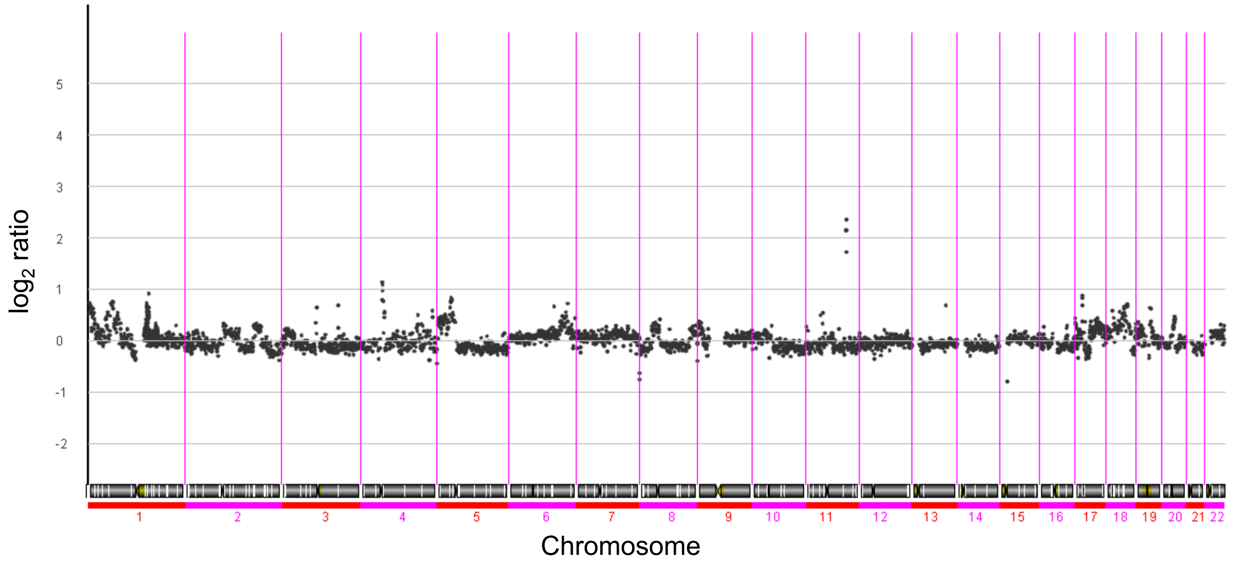

MFH73x

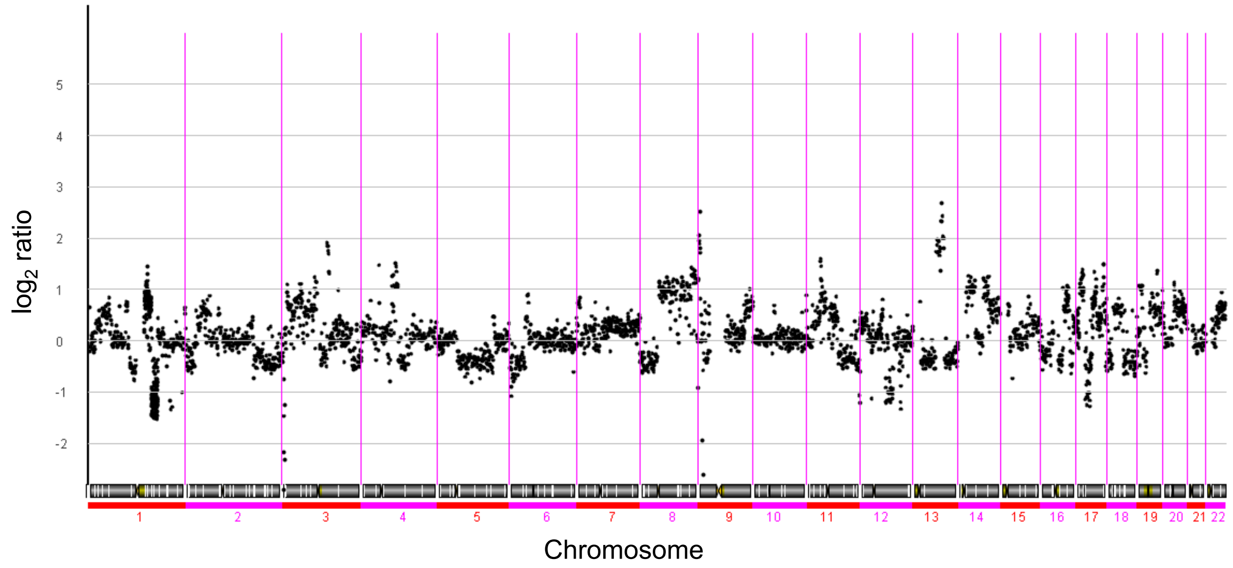

MFH76x

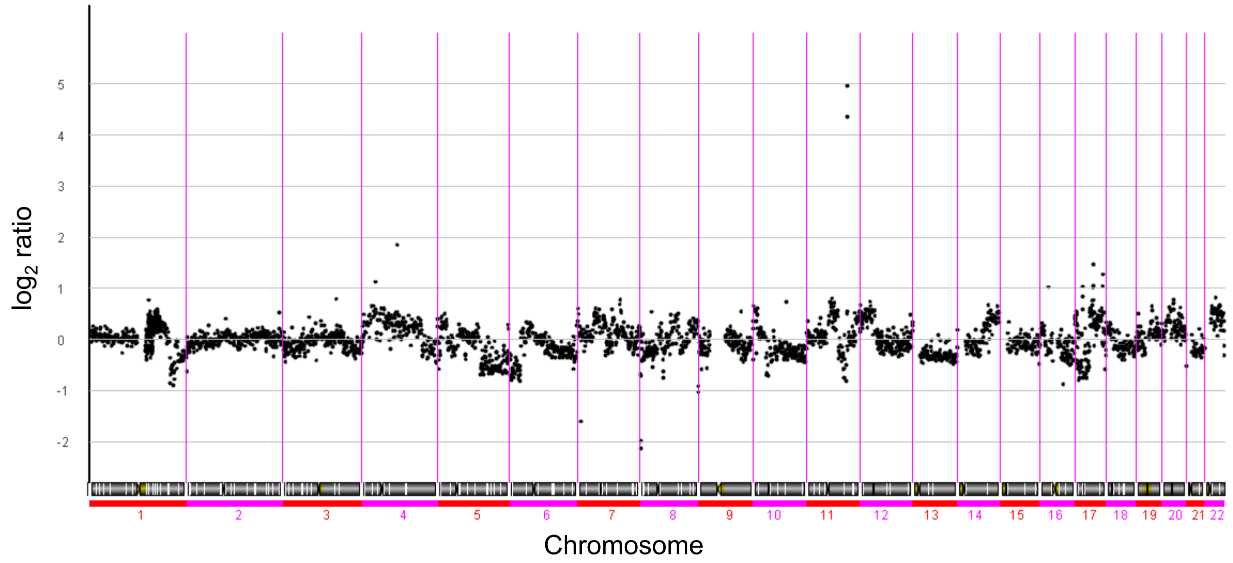

Supplement: Figure S1 — Genome-wide ratio plots of 33 MFHs. (PDF) [file pone.0015378.s001.pdf]
