# Supplementary figures and images for: DNA Copy Number Changes in Human Malignant Fibrous Histiocytomas by Array Comparative Genomic Hybridisation
Source: PLoS One. 2010 Nov 9;5(11):e15378. doi: 10.1371/journal.pone.0015378 (PMC2976768; doi:10.1371/journal.pone.0015378)

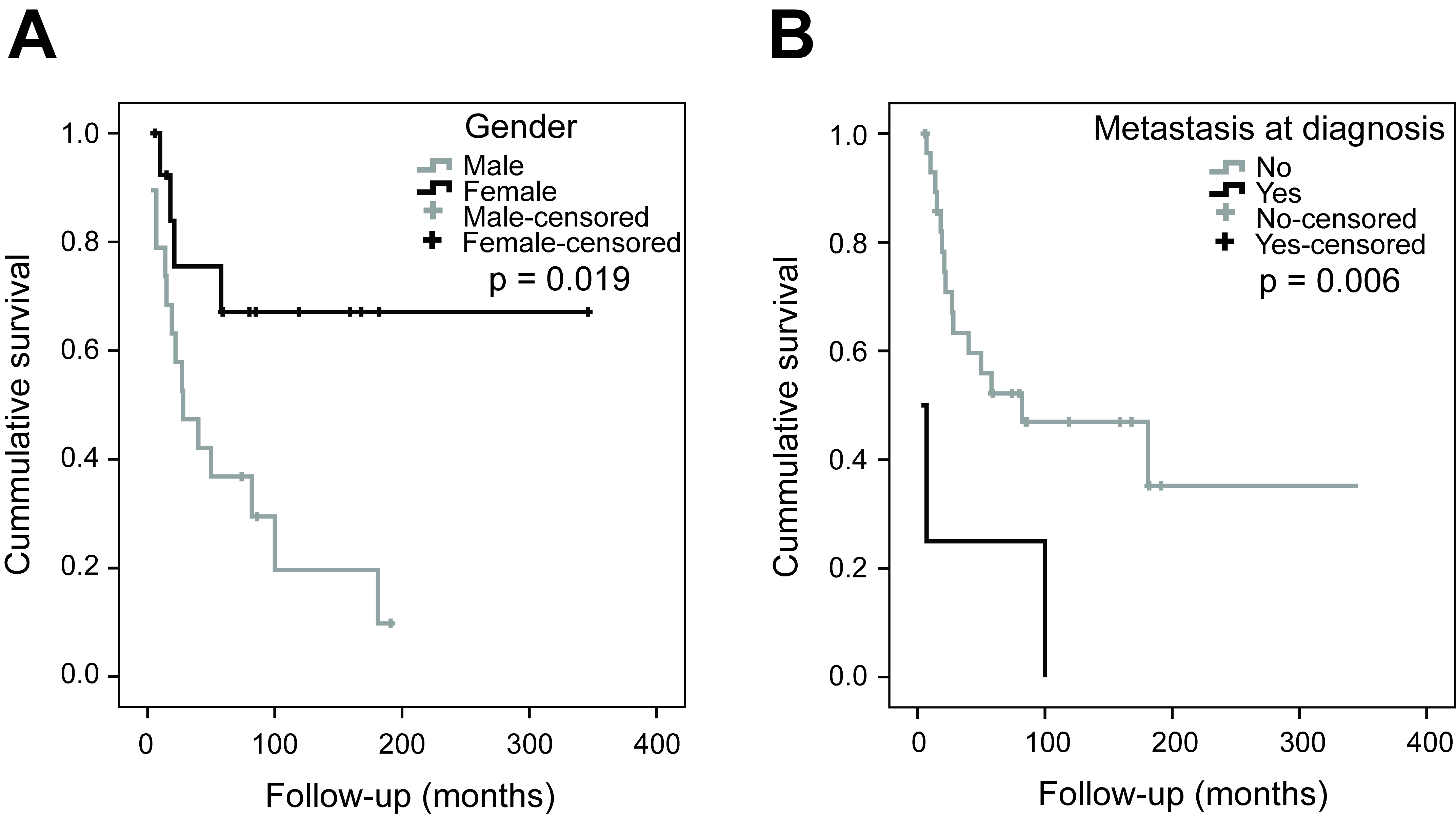

Supplement: Figure S2 — Kaplan-Meier plots with overall survival curves for A) female patients (n = 14) and male patients (n = 19) and B) patients with metastasis at diagnosis (n = 4) and patients without (n = 29). (TIF) [file pone.0015378.s002.tif]
